# Arsenic efflux and bioremediation potential of Klebsiella oxytoca via the arsB gene

**DOI:** 10.1371/journal.pone.0307918

**Published:** 2025-01-29

**Authors:** Sana Waqar, Aamira Tariq, Ubaid ullah, Hira Haleem, Hadiqa Aimen, Sadia Sattar, Nazish Bostan

**Affiliations:** 1 Microbiology and Public Health Labs, Department of Biosciences, Comsats University Islamabad, Islamabad, Pakistan; 2 Molecular Virology Labs, Department of Biosciences, Comsats University Islamabad, Islamabad, Pakistan; St Cloud State University, UNITED STATES OF AMERICA

## Abstract

Arsenic-resistant *Klebsiella oxytoca* strain AT-02 was isolated from the ground water of the Multan region of Pakistan. The strain displayed high arsenite and arsenate resistance as minimal inhibitory concentration (MIC) was 600ppm and 10,000ppm respectively. The high tolerance of the isolated strain towards arsenate can be postulated due to significant increase in biofilm in response to arsenate. The bacterial strain exposed to 1/2 and 3/4 MIC showed a significant 10 and 12 folds increase in expression of the arsenite efflux gene arsB. Sequential and structural comparison of the arsB gene showed the presence of conserved arsenic binding residues. Arsenic remediation by AT-02 biomass was 50% after 0.5 hours of incubation and 66% in 2 hours. the increase in remediation efficiency with the increase in incubation time indicates its biosorption potential. the arsenic sensitive strain NK11 showed only 4–5% arsenic remediation. Fourier transform infrared spectroscopy (FTIR) analysis confirmed interaction of arsenate and arsenite with functional groups (aromatic amino acid residues) on the cell surface leading to characteristic peak shifts. Thus, the isolated AT-02 has the potential to remediate both arsenite and arsenate from contaminated environmental sites.

## 1. Introduction

Developing countries like Pakistan, have arsenic contamination issues. The shallow and deep aquifers of Punjab, Pakistan are observed to be arsenic contaminated mainly due to anthropogenic activity [1] like extensive industrialization and non-regulated use of heavy metal-containing chemicals on agricultural crops leading to the contamination of soil and eventually water bodies. Particularly in south-Punjab arsenic contamination has been documented in different areas like Multan [2–4], and Vehari [2–6]. Arsenic is graded as Class I carcinogen due to its higher affinity towards lipids and fat tissues deposition tendency [7]. The population residing in Multan is at high chronic health risk due to the consumption of arsenic-contaminated water [2]. Bioremediation has gained worldwide recognition for heavy metal removal from contaminated water bodies and soil owing to high efficiency, minimal secondary pollution, and cost-effectiveness [8]. In comparison to plants, offering simple bioaccumulation microbes can alter the toxicity, mobility, and bioavailability through different strategies either in-situ or ex-situ like bio-accumulation, bio-sorption, bio-mineralization, and bio-transformation for detoxification of heavy metals [9, 10]. However, few studies focus on the investigation of resistance mechanisms presented by the well-adapted microbes in such environments and investigate plausible resistance mechanisms [11, 12].

The most common oxidation states of soluble arsenic present in their oxyanion form in the environment are arsenate (As V, AsO_4_), and more toxic arsenite (As III, As (OH)_3_) [13, 14]. Arsenate can form manu unstable intermediates by inorganic phosphate replacement in DNA, RNA and proteins. It also inhibits ATP generation and hampers the ATP dependent metabolic processes and signal transduction pathways. Arsenite (As III) has high affinity towards sulfhydryl and thiol groups in different biomolecules hindering their activity [15]. Bacteria have developed different mechanisms in order to evade arsenic toxicity i.e. peroxidation of membrane lipids, minimizing arsenate absorption by the phosphate uptake system, and arsenic efflux by two different and unrelated families of proteins ArsB and Acr3p [16]. Different bacteria like Brevundimonas nasdae strains, Au-Bre29 and Au-Bre30 excrete extracellular polymeric substance to mediate arsenic toxicity [17]. Similarly *Agrobacterium tumefaciens (*GW4), *Rhizobium* sp. (NT-26), utilize arsenic as source of energy for growth sustainability. arsB gene encodes a membrane-bound permease ArsB that exports arsenite and is predominantly present in the chromosomes of Proteobacteria, Actinobacteria, and Firmicutes [18]. ArsB typically comprises of 12 transmembrane helices with arsenic binding residues located at different regions [19]. The bioavailability of arsenic is also reduced in biofilms that are often formed by bacteria as a stress response to the environment [20]. Keeping in view, the arsenic toxicity and its hazardous impact on human health the removal of arsenic from the environment is imperative. The traditional approaches to remove arsenic from contaminated sites are time consuming, expensive and even hazardous. Eco-friendly approaches like microorganisms based bioremediation has great potential. Bacterial mass can interact with arsenic through various mechanisms like entrapment, precipitation, methylation, oxidation reduction, intracellular absorption and extracellular absorption [21]. Heavy metals adhere to different functional groups like amino, amide, carboxyl, imidazole and sulfonates [22]. A arsenic oxidizing bacterial strain *Proteus alimentor*um TY6 isolated from India are recently reported to remediate arsenic by 66% [23]. However, the underlying molecular mechanism is not revealed in this study.

In the present study, an arsenic-resistant bacterial strain was isolated a from contaminated groundwater in Multan. We further characterized and investigated the underlying resistance mechanisms and possible bioremediation potential of its bacterial biomass.

## 2. Materials and methods

### 2.1 Study area and sampling

Multan city was selected as the study area being the fifth largest city of Pakistan in terms of population with an area 3721 km. It lies on the alluvial planes of Chenab river that serves as the main source of water supply and ground water recharge [24]. Arsenic contamination is well reported in this area in several studies [2, 4, 12]. Ground water sample was collected from hand pump after 5 min flushing of wells to ensure fresh sample collection. The sample was taken in a sterilized plastic tube and kept at 4°C until further use.

### 2.2 Physio-chemical analysis of water sample

After the collection of the water sample, physio-chemical analysis was performed to measure and check the pH, electrical conductivity, total dissolved solids (TDS), acidity, alkalinity, hardness, and salt contents in the water. pH and electrical conductivity were measured using a pH meter (Hanna Instrument model 8519, Italy) and a digital conductivity meter (E.C. meter Hach-44600-00, USA) respectively. Turbidity (NTU) was determined by a turbidity meter (Lamotte, Model 2008, USA). Total dissolved solids (TDS) were measured in mg/L by using a TDS meter (Model 2540C, standard method, 1992). Total alkalinity was determined using acid titration, with methyl orange as an indicator (2320, standard method, 1992). Calcium hardness, Mg hardness, and total hardness, as CaCO3, were determined as reported previously [25, 26]. Chloride was estimated by potentiometric titration with a standard 0.0141N silver nitrate solution (standard method,1992). Sulfate ions were precipitated as barium sulfate in an acidic medium with barium chloride. The absorption of light by the precipitated suspension was measured via spectrophotometer (SulfaVer4 (Hach-8051) at 420 nm (APHA, 22nd edition). The cations and heavy metals were analysed using the flame atomic absorption spectrophotometer (AAS Vario 6, Analytik Jena AG) method (Kopp and McKee1979). Samples were left unfiltered to provide accurate values.

### 2.3 Isolation and biochemical characterization of arsenic resistant strain

Water samples (120–140 ml) were filtered through 2μm syringe filter and later the filter membrane was placed in 100 ml LB broth media having 50 ppm (μg/ml), Arsenate (Sodium Arsenate (Na_2_HAsO_4_.7H_2_O) Cat # S9663 Sigma-Aldrich) and Arsenite (Sodium meta-arsenite (Na_2_AsO_2_) Cat # S7400 Sigma-Aldrich). It was then incubated in the shaking incubator for 24 hours at 37°C. After overnight incubation 10 ml medium was taken from this culture and inoculated in 90 ml LB broth, supplemented with 200 ppm (μg/ml) arsenite and 1000 ppm Arsenate for 24 hours time period at 37°C. Next day 10μL sample was spread on LB agar plates and incubated at 37°C overnight. Following day individual colonies were selected and streaked on the LB agar plates with different concentrations of As^3+^ (200–800 ppm (μg/ml) and As^5+^ and (1000 to 10, 000 ppm μg/ml). The selected colonies capable of growing at higher concentration of arsenic were selected and stocked. Later this strain was identified as *Klebsiella* by catalase, oxidase and TSI tests as well as fermentation of carbohydrates and gram staining. Molecular characterization was done by amplification and NCBI BLAST homology analysis of the 16SrRNAsequence of The strain using universal primers (27F 5’-AGAGTTTGATCCTGGCTCAG-3’; 1492R 5’-GGTTACCTTGTTACGACTT-3’) [27]. The amplified product was subjected to Sanger sequencing. The obtained sequence was aligned with the non-redundant sequence database at NCBI using BLASTn (https://blast.ncbi.nlm.nih.gov/Blast.cgi?PROGRAM=blastn&BLAST_SPEC=GeoBlast&PAGE_TYPE=BlastSearch). BLAST n analysis identified this strain as *Klebsiella oxytoca* and named as AT-02.

### 2.4 Evaluation of arsenic tolerance by AT-02

The growth curve of AT-02 in presence of arsenate ((Na_2_HAsO_4_.7H_2_O) Cat # S9663 Sigma–Aldrich) and sodium ((Na_2_AsO_2_) Cat # S7400 Sigma–Aldrich) was obtained by inoculating the strain in LB medium containing various concentrations of arsenate (1000 ppm- 10,000 ppm) and arsenite (200 ppm-800 ppm (μg/ml)) separately. The cultures were allowed to grow at 37°C for 24h. Every 2h a sample was removed, and growth progression was checked by measuring OD at 600nm. Tolerance of Arsenate and arsenite was also tested by plating the strains on solidified LB agar plates with various concentration (1000–10,000 ppm and 200–800 ppm respectively) of both. Briefly an exponential culture of AT-02 was taken, and OD 0.2 and 10-fold serial dilutions were prepared. These dilutions were plated on solidified agar plates carrying different concentrations of Arsenate and arsenite. Plates were incubated at 37°C for 24h.

### 2.5 Determination of arsenic tolerance mechanism by AT-02

To identify mechanism of arsenic resistance arsB gene, a member of ars operon was amplified using following primers F5’ATGCTACTGGCAGGTGCTATT3’ and R 5’ TCATGACAAAGTGAAAGAGAGACGTAG 3’. The amplified product was sequenced and analysed by DNA and Protein homology tools to find it closest neighbours. The nucleotide sequence was translated using EXPASY translated tool freely available at (https://web.expasy.org/translate/), later the sequence was analysed with BLAST p to find its closest relatives in NCBI. To find amino acid homology a multiple sequence alignment was performed using T-Coffee (https://www.ebi.ac.uk/Tools/msa/tcoffee/) and MUSCLE (http://www.ebi.ac.uk/Tools/msa/muscle/) A neighbour joining phylogenetic tree was generated with 16SrRNA sequence of AT-02 and ArsB gene from AT-02 and its close homologs using MEGA11 software [28]. For nucleotide, phylogenetic tree sequence of 6 close homologs was used whereas 12 close homologs were used to generate phylogenetic tree using protein sequences. The percentage of replicate trees in which the associated taxa are clustered together in the boot strap test comprising of 500 replicates are shown next to branches [29]. Evolutionary distances were computed by Maximum Composite Likelihood Method in units calculated as number of base substitutions per site [30]. Poisson correction method was adopted for the computation of evolutionary distances in the case of ArsB protein sequences (Fig 3A). All positions containing gaps and missing data were eliminated. For the ArsB protein sequences with distant homologs, Maximum likelihood method and JTT matrix based model [31] was used for phylogenetic analysis comprising of 6 protein sequences. Maximum likelihood method was employed in which first tree for heuristic search was obtained by applying Neighbour–Join and BioNJ algorithm to a matrix of pairwise distances using JTT model followed by the selection of superior log likelihood value. There was a total of 707 positions in the data set (Fig 3B).

### 2.6 Protein structure prediction and analysis

The arsB amino acid sequences of *K*.*oytoca*, *E*.*coli*, *K*.*pneumoniae* and *P*.*putida* were further subjected to Three-dimensional structure prediction using trRosetta [32] and LOMETS [33]. The predicted structures were further validated using ERRAT [34] and PROCHECK [35]. The structures were superimposed using TM-align [36]. Mutational analysis of ArsB arsenic binding residues was done by PROVEAN [37] and DDMUT [38].

### 2.7 Differential expression analysis for efflux pump protein arsB in response to arsenite

The induction of the arsB gene in response to presence of arsenite in the medium was determined by growing AT-02 strain with arsenite for 18h. This value was compared to the amount of ArsB gene expression when grown in LB without arsenite. The final concentrations of arsenite used in this experiment were 0.25, 0.5 and 0.75 times of its MIC. Briefly total RNA was extracted from AT-02 strain using Trizol method (Invitrogen Cat #15596026) grown on three different concentrations of sodium arsenite (200 ppm, 400 ppm, 600 ppm). AT-02 strain grown without arsenite was used as control. Revert Aid cDNA synthesis kit (Thermo-Scientific Cat # K1691) was used to synthesize cDNA from extracted RNA. Differential Expression was quantified by quantitative real time PCR with Thermo-Scientific Maxima Sybr Green (Cat # K0221). For real time PCR analysis primers were synthesized for arsB using NCBI primer blast interface (https://www.ncbi.nlm.nih.gov/tools/primer-blast/). All primers are listed in S1 Table. Real time qRT-PCR analysis was carried using ABI step-one system and the 16SrRNA was used for normalization of gene expression as described by [39]. The assay was carried out in triplicates and results were document by comparing relative change between induced and non-induced samples with and without arsenic respectively. The relative change in expression of the arsB between the induced and non-induced samples was calculated using the 2^−ΔΔCT^ as described by Livak and Schmittgen [40].

### 2.8 Biofilm assay

AT-0 2 was allowed to grow in presence of sodium arsenate (1000 ppm μg /ml) in shaking incubator at 37°C for 24h. The same strain grown in LB without Arsenate was used as control. Briefly, an overnight culture was diluted to 1:1000 fold to get an OD of 0.1 at 600 nm and 200 μL were transferred to a clean test tube and incubated at 37°C for 48h [41]. After 48h of incubation, OD of supernatants was measured at 600 nm and was removed. The empty tubes were washed 3 times with distilled water. 200 μl of 10-fold crystal violet (CV) dilution was added to all test tubes containing dry biofilms. After 1hour crystal violet was removed and tubes were washed three times with distilled water. Finally, the fixed CV was dissolved with 200 μl DMSO. The absorbance of solution was detected at 560 nm on spectrophotometer. The significance of difference was tested by comparison of OD with control sample. The experiment was run in triplicates. The OD of control (ODc) was calculated with optical density (OD) values of negative control i.e. plain LB [41].

### 2.9 Arsenic bioremediation by AT-02 biomass

Bacterial biomass was prepared to test the ability of bacterial strains AT-02 and NK11to remove arsenic from the growth environment. The biomass of AT-02 and NK11was obtained by the inoculation of 25 ml overnight culture in 500 ml of LB broth medium. Incubation was carried out at 37°C for 18 to 24 hours. The culture was centrifuged 4000 rpm for 20 mins after incubation and washed multiple times with deionized water to remove impurities. Pellet was dried in the oven at 100°C overnight. Next day, the bacterial biomass was collected in the form of powder. Same procedure was repeated 8–10 times to get biomass of bacteria in sufficient amount [42]. Further arsenic remediation was tested using biomass of the respective bacteria. Briefly, master stock of both Arsenate and arsenite (10,000 ppb) was diluted to 100 ppb (parts per billion) and adjusted to pH 7. Next bacterial biomass (AT-02) was added to a concentration of 1g/L in each tube. Temperature was maintained at 37°C and shaking at 133 rpm. An aliquot of 50 ml was collected from each tube every 30 mins till 2h. After 2h remaining solution was filtered and biomass was separated. Filtrate was then acid digested followed by Atomic Absorption Spectroscopy (AAS) to check the remediation of arsenic. The remediation efficiency was calculated as described previously by [42]. The remediation efficiency was determined by using the following formula

$$E=(\frac{C_{i}-C_{f}}{C_{i}})*100$$

Where Ci is the initial concentration of the metallic ion (mg L-1), Cf is the final concentration of the metallic ion (mg L-1), m is the mass of the biosorbent in the reaction mixture (g), and V is the volume of the reaction mixture (L)

### 2.10 Fourier–transform infrared spectroscopy (FTIR) analysis of arsenic adsorption

All of the chemicals were procured from Sigma Chemicals Co. (St. Louis, USA). Stock solutions of arsenate ((1000 mg/L) by dissolving 4.16 g of Na2HAsO4.7H2O in 1.0 L of de-ionized water)) and arsenite ((As+3) 1000 mg/L was prepared by dissolving 1.73 g of NaAsO2 in 1.0 L of 0.1% ascorbic acid solution). Arsenic-loaded biomass for Fourier transform infrared (FT-IR) analysis was prepared by incubating 1 g/L of dried biomass with 50 mL of 100 mg/L As^+3^ at pH 7.0 and As^+5^ at pH 3.0 for 60 min. A disc of 100mg KBr containing dried 1%AT-02 cells served as the material for recording transmission spectra. Spectra were recorded in the range 400–4000 cm−1 using a FT-IR spectrometer (Spectrum GX, Perkin Elmer, USA) with a resolution of 0.15 cm−1 to evaluate functional groups that might be involved in the sorption process.

### 2.11 Statistical analysis

The remediation, biofilm formation, and real-time expression analysis experiments were done in triplicates. Their mean and standard deviation were calculated. Student t-test was used to evaluate the significance of the data. A P-value less than 0.05 was considered significant.

## 3. Results

Arsenic resistance is a growing issue all around the world. Arsenic contamination in drinking water is related to several health hazards and its bioremediation is much easier than its chemical removal. This study was designed in the similar perspective.

### 3.1 Isolation and characterization of bacterial strain with remediation potential

Multan district is one of the largest districts directly/indirectly associated with agricultural and industrial development in Pakistan. This region lies adjacent to the country’s largest cotton and wheat bed. It is well established that water in Multan district is contaminated and has a high prevalence of arsenic in it [4, 43] causing human health hazards. Therefore, a sample of groundwater was collected from this region had a high chance of arsenic-resistant bacteria. The collected ground water exhibited a pH 7.3 (S2 Table) and a temperature range of 26–30°C. The slight alkaline condition might result in arsenic release in the groundwater via soil.

This water was arsenic contaminated resulting in the release of arsenic-resistant bacterial species. One such sample labelled as AT-02 tested positive for bacterial growth at high concentrations of arsenic.

### 3.2 Molecular and biochemical characterization of the bacterial strain

**Fig 1:**
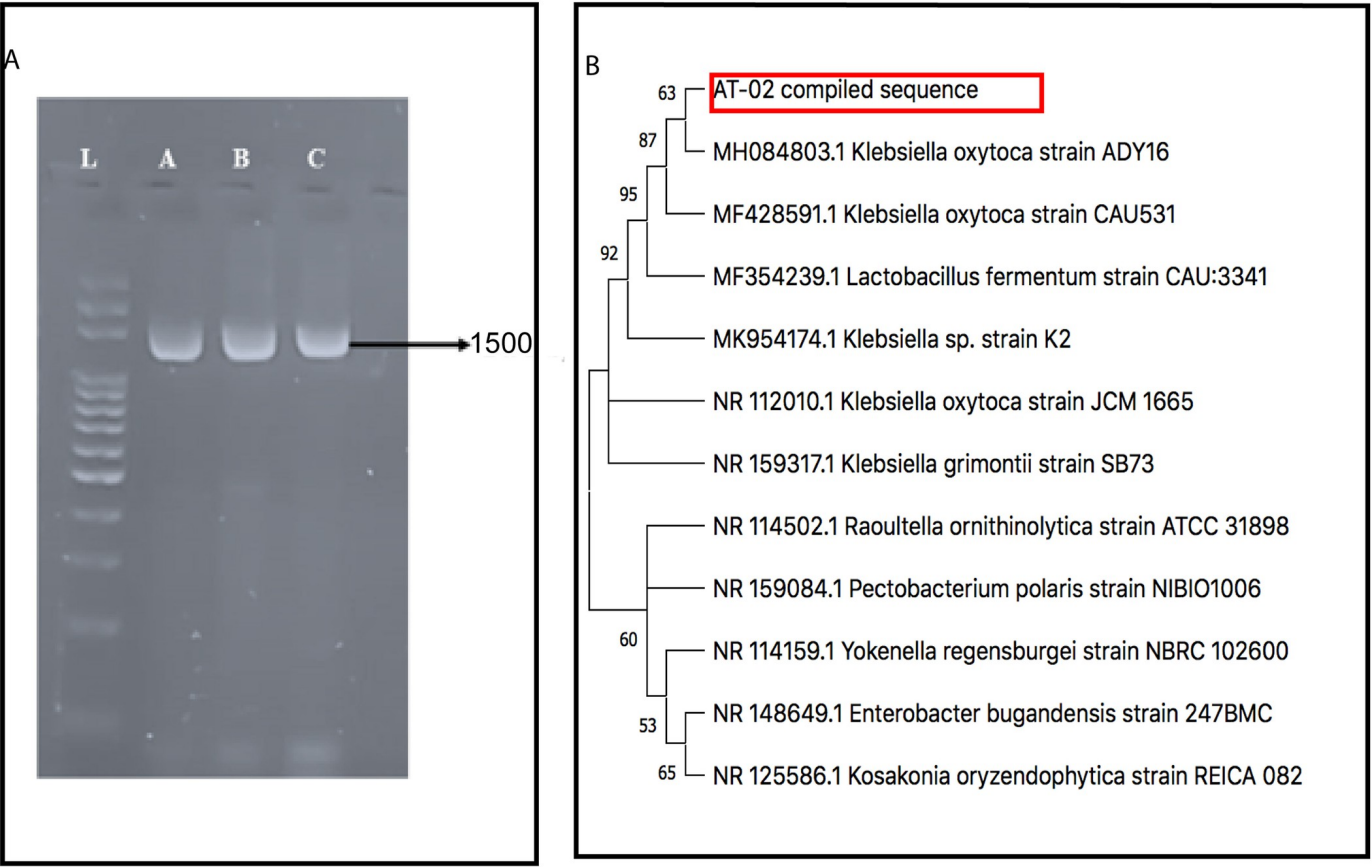
Molecular characterization of arsenic resistant strain. **(A).** PCR amplification of the isolated strain AT-02 DNA (using different dilutions) via universal 16SrRNA primers 27F and 1492 R. Lane 1,2, and 3 shows the amplified band of 1500b.p by the universal primers. The amplified product was further subjected to sequencing. **(B).** Phylogenetic tree constructed via Neighbour Joining method using MEGA11 showing high similarity of the isolated AT-02 strain with *Klebsiella oxytoca ADY16* strain.

The biochemical identification of the bacterial isolate capable of growing in a high arsenic environment was identified as a Gram-negative, rod labelled as AT-02. Biochemical tests were carried out for the identification of this bacterial species. These results indicate that the bacterial strain was oxidase-negative, catalase, indole, citrate and Triple sugar iron (TSI) test positive (S1 Fig). These biochemical tests, morphological features, and gram-staining characteristics indicate that AT-02 belongs to the genus *Klebsiella*. Further confirmation of the strain was performed by amplification of 16SrRNA using the universal 27F and 1492R primers (Fig 1A) as *Klebsiella oxytoca* due to its resemblance with a heavy metal resistant strain isolated from soil *K*. *oxytoca* ADY16 strain in NCBI, BLAST. In order to find the phylogenetic relation of AT-02 with other homologs in BLAST a tree was constructed using the Neighbour joining method [44] using MEGA11 [28] (Fig 1B). The percentage of replicate trees in which the associated taxa are clustered together in the bootstrap test comprising 500 replicates are shown next to branches [29] as depicted in Fig 1B. Evolutionary distances were computed by Maximum Composite Likelihood Method in units calculated as the number of base substitutions per site [30].

### 3.3 Minimum Inhibitory Concentration (MIC) and Maximum Tolerable Concentration (MTC) of AT-02

**Fig 2:**
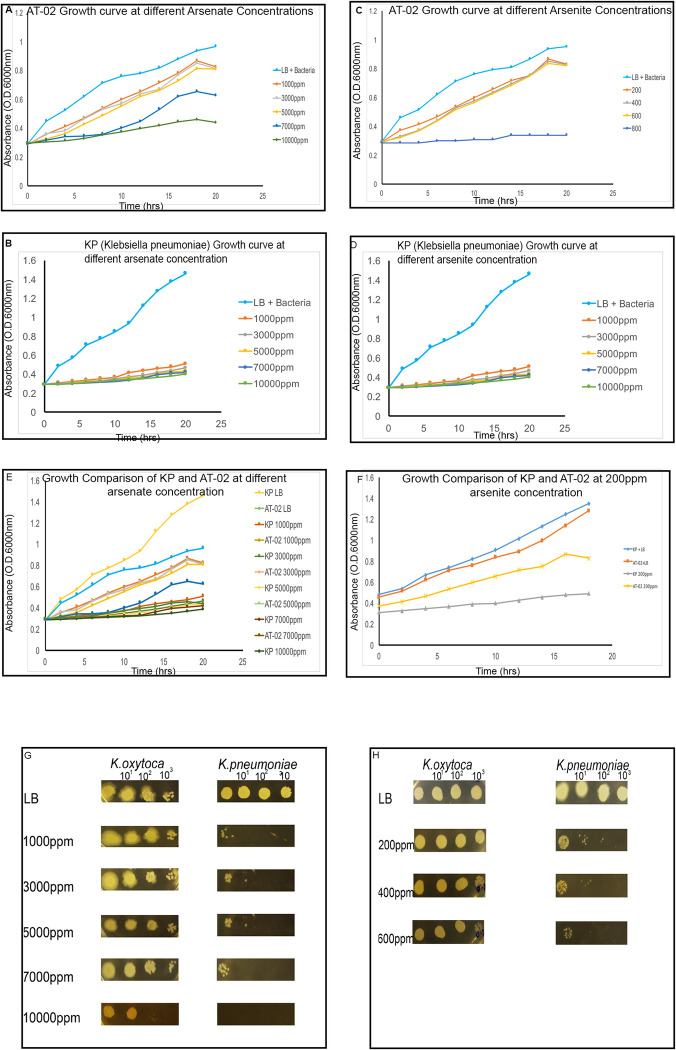
Determination of the AT-02 strain arsenic resistance. **(A).**Growth curve of the AT-02 strain at different arsenate concentrations ranging from 1000–10,000 μg/mL showing that the isolated strain can survive till 10,000 μg/mL. Media without arsenate (shown in blue) is indicated as LB was used as a control. **(B).**Growth curve of the AT-02 strain at different arsenite concentrations ranging from 200–600 μg/mL showing that the isolated strain can survive till 600 μg/mL. Media without arsenite is indicated as LB (shown in blue) was used as a control. **(C).**Growth curve of the reference strain *Klebsiella pneumoniae* strain at different arsenate concentrations ranging from 1000–10,000 μg/mL showing arsenic sensitivity. The control strain can only grow easily in the absence of arsenate. **(D).**Growth curve of the *Klebsiella pneumoniae* strain at different arsenite concentrations ranging from 200–600 μg/mL μg/mL showing that the reference strain was not able to survive in the presence of arsenite. **(E&F).**Growth comparison of *Klebsiella oxytoca* (AT-02) with *Klebsiella pneumoniae* on LB broth bearing different concentrations of arsenate (1000–10,000 μg/mL) and arsenite (200 μg/mL). **(G&H).** Growth comparison of *Klebsiella oxytoca* (AT-02) with *Klebsiella pneumoniae* on LB agar plates bearing different concentrations of arsenate (1000–10,000 μg/mL) and arsenite (200–600 μg/mL). The comparison showed that the *Klebsiella oxytoca* (AT-02) can survive till 10,000ppm arsenate and 600ppm arsenite respectively.

The lowest concentration of arsenate that can inhibit AT-02 growth (MIC) and the maximum concentration that AT-02 can tolerate (MTC) was determined by incubating the isolated strain in LB containing sodium arsenate (1000–10,000ppm) (μg/ml) and sodium arsenite (200-600ppm) (μg/ml). The growth curves were plotted at different concentration of arsenate and arsenite based bacterial growth determined by optical density (O.D). A clinical isolated K. pneumoniae ((Kp) NK11 documented previously [45] was used as negative control for arsenic resistance. Both AT-02 and negative control had 4h lag phase in both arsenic rich and no arsenic environment (Fig 2A–2D). In the presence of arsenic a strong decrease in growth was observed in the *K*.*pneumoniae* (Kp) strain. However, the isolated arsenic resistance strain showed progressive increase in growth at sodium arsenate (1000–5,000ppm) (μg/ml) and sodium arsenite (200-600ppm) concentration respectively. Growth comparison between AT-02 and Kp showed a significant reduction in growth in the case of KP at different arsenate concentrations with a P-value of 0.00463455, 0.001915362, 0.002799637, 0.022699788 and 0.003181837 at 1000,3000, 5000,700 and 10,000ppm) (μg/ml) respectively. On the contrary, in the presence of arsenite significant growth reduction (P-value 0.001176208) was observed in the case of Kp as opposed to the AT-02 strain (Fig 2E & 2F). Thus, AT-02 strain showed stronger resistance towards arsenate as compared to sodium arsenite.

The growth curve results were further validated by growing the bacteria in dilution series on LB- agar plates with and without arsenic. The strains grew well on LB plates without arsenic. However, strong decline in growth was observed in case of Kp due to the presence of arsenic. AT-02 showed similar resistance pattern on plates with varied amount of arsenic (Fig 2G and 2H). AT-02 can tolerate approximately 10,000ppm (μg/ml) arsenate and 600ppm (μg/ml) arsenite respectively. AT-02 growth was faster at lower concentrations of arsenate and arsenite. It dropped as concentrations increased.

### 3.4 Amplification of arsB

**Fig 3:**
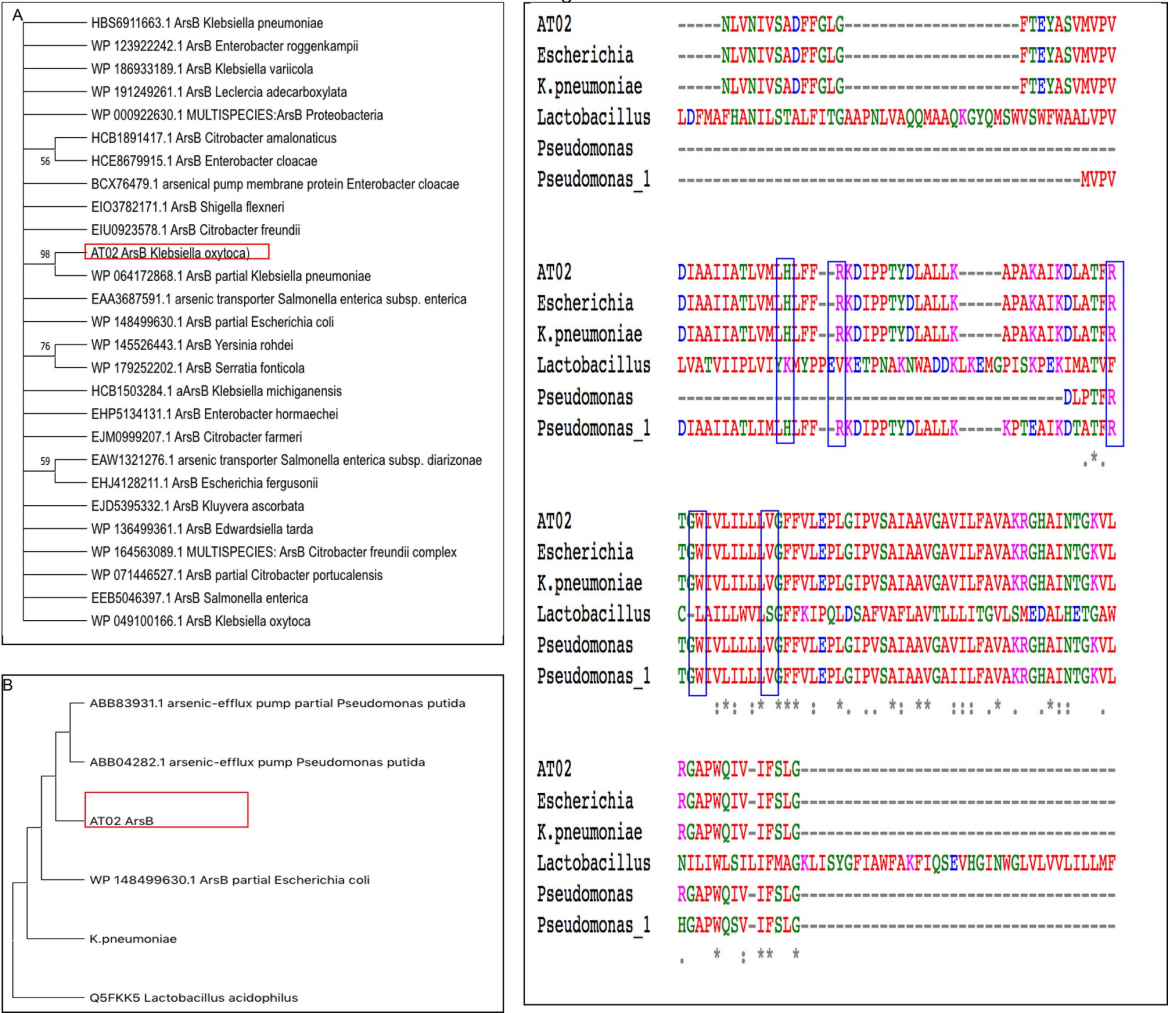
Amplification and sequence analysis of arsB. **(A).** Phylogenetic tree based on Neighbour joining method using MEGA11 based on 27 amino acid sequences showed similarity between arsB of *AT-02* and *Klebsiella pneumoniae*. **(B).**Phylogenetic tree between distantly similar species was constructed by Maximum Likelihood method showed similarity between arsB of *AT-02* and *E*.*coli*. **(C).**Multiple sequence alignment based on 6 different amino acid sequences show presence of conserved arsenic binding residues (Histidine (H), Arginine (R), Arginine (R), Tryptophan (W), Valine (V)) highlighted in blue boxes. These residues are conserved in *AT-02*, *E*.*coli*, *K*. *pneumoniae* and *P*.*putida*, *However*, *the in Lactobacillus* these residues are replaced by Lysine (K), Valine (V), Phenylalanine (F),Leucine(L) and Serine (S).

To determine the resistance acquisition method of AT-02 towards arsenite, the arsenic efflux pump gene arsB was amplified. The nucleotide sequence was translated and the respective ORF was used for phylogenetic analysis. Sequence alignment of 27 amino acid sequences was done by using MUSCLE followed by phylogenetic tree generation using MEGA11. Phylogenetic tree based on Neighbour Joining method [44] showed AT-02 arsB resemblance with the arsB of *K*.*pneumoniae* partial sequence (WP064172868.1) (Fig 3A). The full protein sequence and its respective Three-dimensional structure was unknown therefore obtained protein sequence was compared with the documented arsenic binding proteins from *Pseudomonas putida* (ABB04282.1, ABB83931.1) [19]. Phylogenetic tree analysis based on Maximum likelihood method [31] showed close resemblance with *E*. *coli* arsB Fig 3B. Multiple sequence alignment with the *P*.*putida*, *K*.*pneumoniae* and *E*.*coli* showed conserved amino acids with putative arsenic binding potential as depicted in the Fig 3C. The putative arsenic binding residues are shown in blue boxes as documented previously for an arsenic resistant *P*.*putida* OS15 stain [19]. The MSA showed presence of conserved arsenic binding residues Histidine (H), two Arginine residues (R), Tryptophan (W) and Valine (V) in the isolated strain AT-02, *K*.*pneumoniae* and *E*.*coli* when compared with the arsB of *Pseudomonas putida* (ABB04282.1, ABB83931.1) Fig 3C. However, the *Lactobacillus* on MSA showed different residues like Lysine (K), Valine (V), Phenylalanine (F), Leucine (L) and Serine (S) at the respective positions. The conservation of amino acids demonstrated that the isolated strain AT-02 harbours arsenic binding potential.

### 3.5 Protein structure prediction of AT-02 ArsB

**Fig 4:**
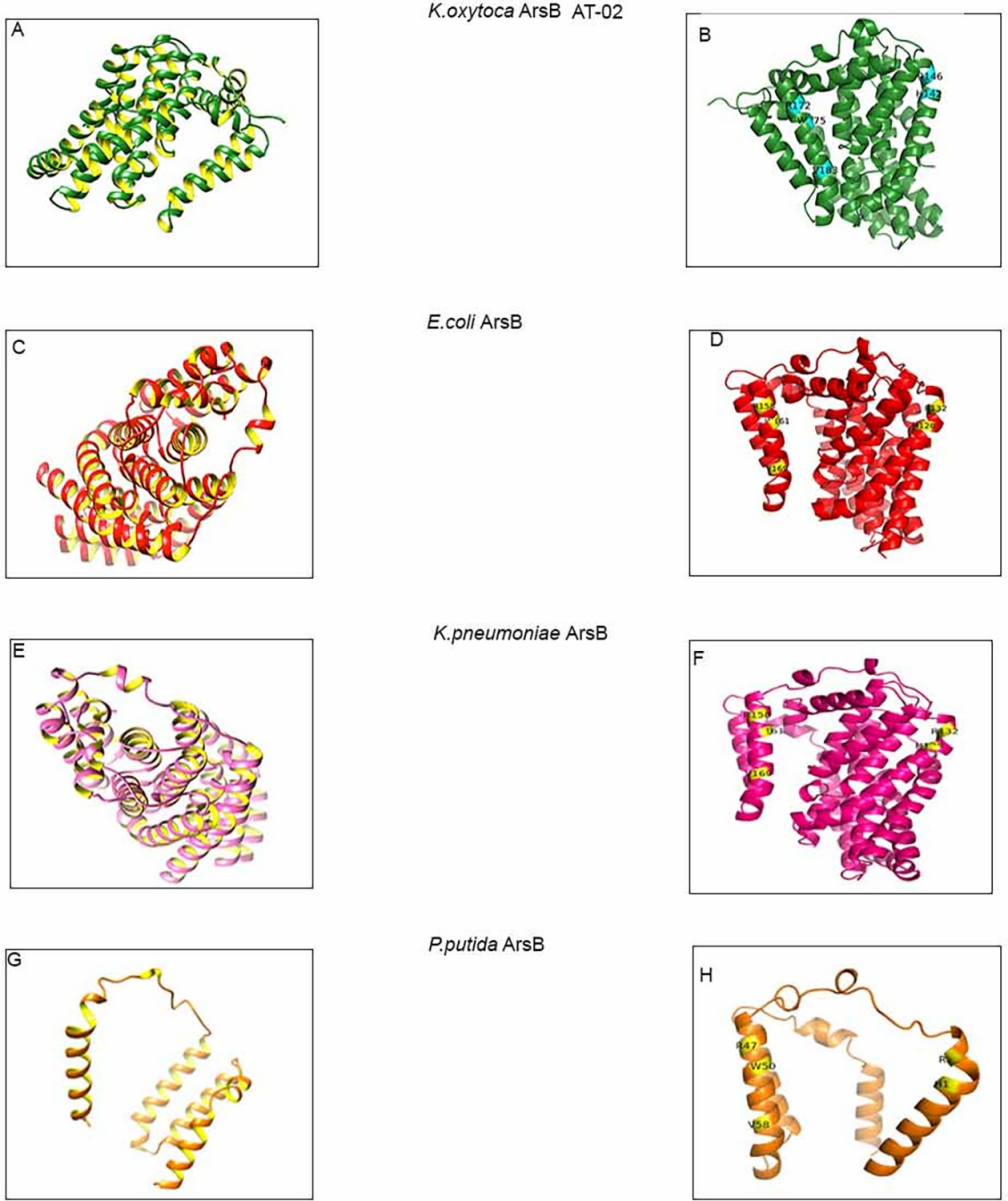
Protein structure prediction of arsB. **(A).** Protein structure prediction of AT-02 (*Klebsiella oxytoca*) ArsB by trRosetta showing 79.8% alpha helix, 4.4% turn, 13.3%coils and 2.5% 3–10 helix. **(B).** Three-dimensional structure of *Klebsiella oxytoca* ArsB with arsenic binding residues highlighted in cyan (Histidine (H142), Arginine (R146), Arginine (R172), Tryptophan (W175), Valine (V183)).**(C).**Protein structure prediction of *E*.*coli* ArsB by trRosetta showing 81.2% alpha helix, 4.4% turn, 13.3%coils and 1.1% 3–10 helix. **(D).** Three-dimensional structure of *E*.*coli* ArsB with arsenic binding residues highlighted in yellow (Histidine (H124), Arginine (R132), Arginine (R158), Tryptophan (W161), Valine (V169)).**(E).**Protein structure prediction of *Klebsiella pneumoniae* ArsB by trRosetta showing 81.2% alpha helix, 4.4% turn, 13.3%coils and 1.1% 3–10 helix. **(F).**Three-dimensional structure of *Klebsiella pneumoniae* ArsB with arsenic binding residues highlighted in yellow (Histidine (H124), Arginine (R132), Arginine (R158), Tryptophan (W161), Valine (V169)). **(G).**Protein structure prediction of *P*.*putida* ArsB by trRosetta showing 72.2% alpha helix, 13.9% turn, 13.9%coils and 0% 3–10 helix.(H). Three-dimensional structure of *P*.*putida* ArsB with arsenic binding residues highlighted in yellow (Histidine (H17), Arginine (R21), Arginine (R47), Tryptophan (W50), Valine (V58)).

**Fig 5:**
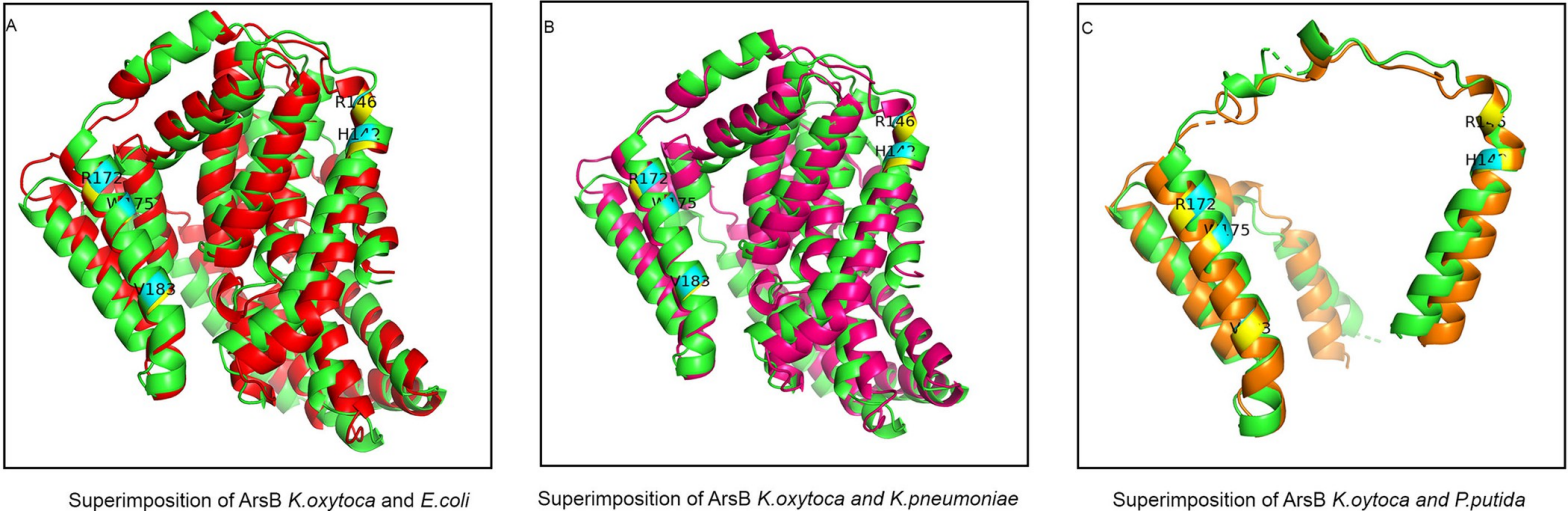
Structural alignment of ArsB. **(A).**Structural superposition of ArsB *Klebsiella oxytoca* and *E*.*coli* using TM-align (https://zhanggroup.org/TM-align/)) showed 96%identical folding indicating high structural similarity with 1.26 RMSD (root mean square deviation) value. **(B).**Structural superposition of ArsB *Klebsiella oxytoca* and *Klebsiella pneumoniae* using TM-align (https://zhanggroup.org/TM-align/)) showed 96%identical folding indicating high structural similarity with 1.26 RMSD (root mean square deviation) value. **(C).** Structural superposition of ArsB *Klebsiella oxytoca* and *P*.*putida* using TM-align (https://zhanggroup.org/TM-align/)) showed 91.3%identical folding indicating comparatively less structural similarity with 2.06 RMSD (root mean square deviation) value.

The high conservation of arsenic binding residues on alignment further urged to determine whether these arsenic binding residues lie in structurally conserved regions. In order to address this query, the obtained protein sequence was further subjected to homology modelling for Three-dimensional structure prediction using trRosetta [32] and LOMETS [33] (Fig 4A). The predicted models were evaluated based on ERRAT [34] and PROCHECK [35]. The best scoring model of the three-dimensional structure of ArsB comprised of mainly helices was predicted by trRosetta based on a plasma membrane transporter template 6wtwA with 97% query coverage and 27.6% sequence identity (Fig 4A). Stereochemistry analysis showed 97.1% residues in the most favoured region. 2.2% in additional allowed region and 0.3% in generously allowed and disallowed region. The overall quality of the predicted structure based on ERRAT analysis was 99.7% indicating a good quality structure. Moreover the ArsB Three-dimensional protein structures were predicted of *E*.*coli*, *K*.*pneumoniae* and *P*.*putida* (Fig 4C, 4E and 4G). The predicted models were evaluated using PROCHECK for stereo-chemical analysis. The predicted models had 98.4%, 98.4% and 99%, residues in the most favoured region. 1.3%, 1.3%, and 0%in additional allowed region, 0% residues in generously allowed region, 0.3%, 0.3% and 0% residues in and disallowed region (S2 Fig). The overall quality of the predicted models based on ERRAT analysis was 99.71%, 99.71%,and 97% respectively indicating high quality of the structures (S3 Fig). The critical arsenic binding residues (Histidine (H), two Arginine residues (R), Tryptophan (W) and Valine (V)) are indicated in cyan for AT-02 (Fig 4B) and yellow in the case of *E*.*coli*, *K*.*pneumoniae* and *P*.*putida* (Fig 4D, 4F and 4H). Superimposition of AT-02 ArsB (shown in green) with the ArsB of *E*.*coli* (shown in red), *K*.*pneumoniae* (shown in pink), and *P*.*putida* (shown in orange) showed the presence of arsenic binding residues in structurally conserved regions indicating that these proteins might bind to arsenic similarly (Fig 5A–5C, S4 Fig). Mutation analysis of critical arsenic binding residues (H142, R146, R172, W175, V183)was carried out using PROVEAN [37] and DDMUT [38]. The results predicted by PROVEAN showed R146A, R172A, W175A as deleterious mutations whereas (H142A&V183A) were neutral mutations having no significant impact on protein stability (S3 Table). Dramatic decrease in the PROVEAN score -12.072 was observed in the case of W175A (S4 Table). DDMUT also predicted W175A as the most destabilising mutation (S4 Table).

### 3.6 Expression analysis of AT-02 arsB

**Fig 6:**
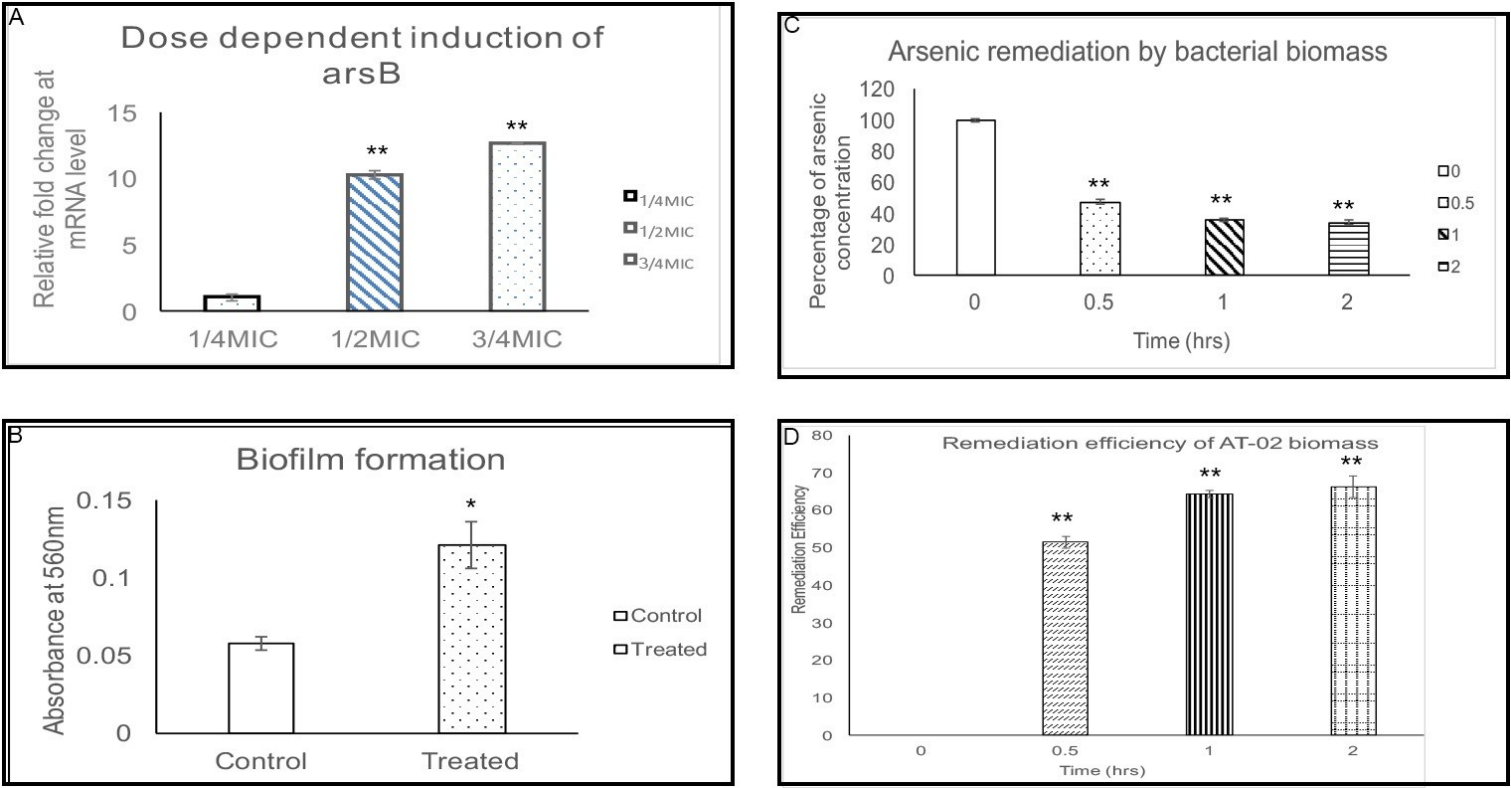
Mechanism of arsenic resistance and bioremediation. **(A).** Real time PCR analysis showing dose dependent increase in arsB expression at RNA level. As compared to ¼ MIC, 1.2 and 3/4MIC showed an increase in arsB by 10 and 12 folds. **(B).** An increase in biofilm formation was observed when AT-02 was grown in the presence of 1000ppm arsenate. A two-fold significant increase in absorbance was observed due to more biofilm formation due to arsenate**. (C).** Biosorption of arsenic at different time intervals showing approximately 50% arsenic removal within 0.5hrs. A slow but increase in arsenic bioremediation was observed till 66% on increasing the incubation time till 2hours. **(D).**The bioremediation efficiency of bacterial biomass against arsenic showed a progressive increase in its removal with increasing incubation time.at different time intervals showing approximately 50% arsenic removal within 0.5hrs. **(A-D).**Error bars represent ±SD of three independent experiments i.e. biological replicates. P-values (*P<0.05, **P<0.01) were obtained by using Student ‘s t-test.

The arsenic binding potential of the ArsB was further tested by inducing its expression in the presence of arsenite. AT-02 was cultured in the presence of different concentrations of arsenite (200-600ppm). The expression level of arsB was compared with the cultures without arsenite using real time PCR. An induction of approximately 10 and 12 folds in arsB expression at RNA level has been observed at 0.5 times and 0.75 times MIC (Fig 6A). The increase in expression of arsB upon elevated arsenite concentration clearly indicates induction of arsB expression in dose dependent manner.

### 3.7 Biofilm formation in response to arsenate

The high resistance of the isolated AT-02 strain towards arsenate intrigued to determine the mode of resistance. Crystal violet staining method was carried out on 48hrs grown biofilms in response to arsenate As(V). The biofilms were cultivated in the media with 1000ppm arsenate (Fig 6B, S5 Fig). Cells firmly attached to the borosilicate tubes were stained with crystal violet. Cells without arsenate produced minimal biofilm whereas presence of 1000ppm arsenate lead to efficient biofilm production. The fixed biofilm stained with crystal violet was dissolved in DMSO and the optical density was observed at 560nm. A twofold increase in optical density in the presence of arsenate indicated increase in biofilm formation (Fig 6B).

### 3.8 Remediation of arsenic by bacterial biomass

The remediation of arsenic by arsenic resistant bacterial biomass and it’s efficiency against remediation of arsenic at different time intervals is shown in the graph Fig 6C. A 100 ppb working solution of arsenic was made for remediation experiment. The percentage of arsenic left after treatment with the bacterial biomass was determined by dividing the amount of unadsorbed arsenic to the initial concentration of arsenic at 0 time point, i.e.,100 ppb (μg/L). The obtained value was multiplied with 100 in order to determine remediation efficiency.

Notable reduction in arsenic level was seen when treated with the biomass of arsenic resistant bacteria. Arsenic’s amount was reduced by 52% in 30 minutes of incubation. The concentration of arsenic was further reduced by increasing the incubation time i.e. in 2 hours of incubation, arsenic concentration was reduced by 66.2%. Thus the bacterial biomass can remove arsenic efficiently. The arsenic sensitive stain NK11 biomass was also tested for arsenic remediation. Interestingly, The NK11 strain showed maximum around 4–5% arsenic remediation efficiency after 2hrs of incubation. The lack of remediation potential in the NK11 strain might be due to the lack of essential functional groups required for arsenic binding (S6 Fig).

### 3.9 Adsorption of arsenic

**Fig 7:**
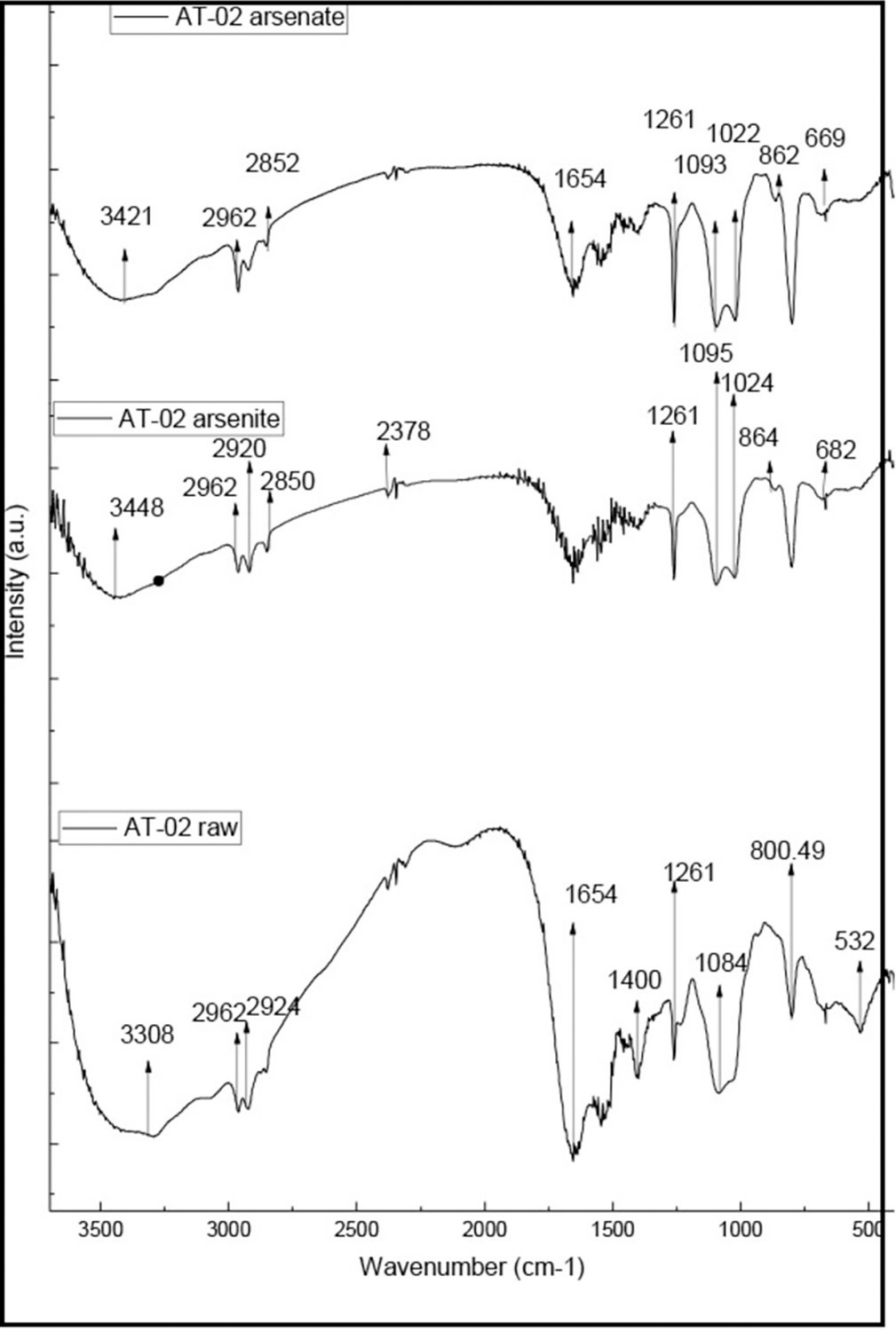
Fourier–transform infrared spectroscopy (FTIR) analysis of arsenic adsorption. FTIR analysis of arsenic adsorption on AT-02 biomass showing peak shifts in the finger print region and above 3000cm^-^.

Fourier–transform infrared (FTIR)–spectroscopy method was adopted to understand the nature of biomass and metal interaction. The spectrum showed the presence of amine, carboxyl, hydroxyl, and aliphatic groups in the biomass. The adsorption of arsenate and arsenite has altered the band intensities of biomass infrared spectra indicating a difference in composition. The broad peak at 3308cm^-1^ is due to the bounded amine (–NH) group in the biomass. The peak at 2924cm^-1^ can be assigned to the aliphatic (-CH) group in the biomass. Peaks at 1654cm^-1^ and 1400cm-1 were attributed to the carboxyl group (-C = O) stretching vibration. However, the bands observed at 1084cm^-1^ were assigned to C-O stretching of alcohols and carboxylic acids. The asymmetrical stretching vibration at 3308cm-1 shifted to 3448 and 3421cm^-1^ for arsenite (As+3) and arsenate (As+5) treated biomass respectively [46]. The peak shift in the fingerprint region from 532cm^-1^ to 682 and 669 for arsenite (As+3) and arsenate (As+5) indicated the involvement of aromatic amino acids for biosorption. Additional peaks are observed at 864, 1024 for arsenite and 862, 1022cm^-1^ for arsenate probably due to As-O stretching vibrations respectively [47]. The peak of the C-O group at 1083 cm-1 shifted to 1095 and 1093cm^-1^ for arsenite (As^+3^) and arsenate (As^+5^) (Fig 7).

## 4. Discussion

Microorganisms tend to play an essential role in the arsenic biochemical cycle by employing different mechanisms like converting to different species via oxidation-reduction resulting in alteration of its solubility, bioavailability, and toxicity [48]. Different studies have reported the isolation of arsenic-resistant bacteria from contaminated groundwater bodies [42, 49, 50]. The groundwater of Punjab and Sindh areas in Pakistan lying along the Indus River basin has high levels of arsenic ranging from 1.5–201 μg/L [51]. The physicochemical analysis of the water sample showed a high amount of arsenic, alkalinity, and bicarbonate levels indicating that the leaching of arsenic might be due to oxidative dissolution. The isolated microbe was able to withstand around 10,000ppm (μg/mL) arsenate and 600ppm (μg/mL) arsenite respectively (Fig 2). This isolated strain was identified as *Klebsiella oxytoca* based on 16SrRNA sequencing (Fig 1). Microbes tend to devise mechanisms to overcome the stress and growth restriction imparted by high arsenic such as cell biomass bio-sorption, cellular capsules entrapment of the heavy metal, and active cell transport by cellular molecules and methylation of arsenic [48, 52]. The microbial resistance towards arsenic is generally attributed either to plasmid or arsRBC operon. Bacteria on exposure to high arsenic concentrations tend to uptake arsenic through their phosphate channels. Cytoplasmic arsenate is reduced to arsenite by a monomeric protein encoded by the arsC gene harboring three critical cysteine residues. The toxic arsenite is extruded through an ATP-driven pump formed by the uniporter protein encoded by the arsB gene [53] in association with ArsA which acts as an ATPase [54]. Generation of arsenite via reduction followed by its efflux using arsB contributes towards arsenic resistance in *Pseudomonas* species [55] and *Campylobacter jejuni [56].* The presence of arsenic detoxification via efflux mechanism was confirmed in the AT-02 strain through successful amplification of the arsB gene. Phylogenetic analysis revealed that the AT-02 ArsB branched with the arsB of *Klebsiella pnuemoniae* 98%, 76% with *Yersinia rohdie* and *Serratia fonticola* and with 59% bootstrap value with *Salmonella enterica* and *Echerichia fergusonii* (Fig 3). Phylogenetic analysis with the well-documented ArsB proteins showed close similarity between the ArsB of *Pseudomonas putida*, and *AT-02* (Fig 3B). Structural analysis displayed putative arsenic binding residues in the AT-02 strain ArsB with *E*. *coli*, *K*. *pneumoniae* and *P*. *putida* ArsB *proteins* (Figs 4 &5). The sequence and structural conservation of arsenic binding residues further support the notion of the arsenic detoxification mechanism employed by the isolated strain. In-silico mutational analysis of critical ArsBarsenic binding residues (H142, R146, R172, W175, V183) to alanine revealed that they can decrease the protein stability which can plausibly effects its functionality (S3 and S4 Tables). The molecular basis of the high resistance offered by the AT-02 strain was further investigated by inducting arsenite at different concentrations followed by expression analysis of the arsB gene. The dose-dependent significant increase in arsB expression at the transcriptional level may address the arsenite resistance offered by the isolated AT-02 strain (Fig 6A). Biofilm formation is a ubiquitous bacterial response to exposure to toxic substances. Biofilm formation by the AT-02 strain on exposure to arsenate might be the plausible reason for its high resistance (Fig 6B).

The biomass of different bacteria like *Bacillus cereus* (6g/L) [57] and *Pseudomonas aeruginosa*(1g/L) [42] have been reported for the bio-sorption of arsenic. Different members of the genus *Klebsiella* have demonstrated heavy metal tolerance [58, 59]. In this study, the biomass of the isolated strain AT-02 was incubated with a known concentration of arsenic (100 ppb). 50% of the arsenic was removed within 30 min. However, a slow increase in arsenic remediation was observed with the increase in incubation time. The complexation of heavy metals with the carboxyl and phosphate groups has been reported as a mechanism of bacteria- based bioremediation [60]. The peak shifts in the fingerprint region and the C-O region demonstrated by the FTIR data further support the arsenic adsorption by the biomass (Fig 7). Different studies have reported the adsorption of arsenic using dried bacterial biomass due to presence of functional groups on the cell surface. FTIR analysis revealed amide, amine and carboxyl groups for arsenate adsorption in the case of Bacillus thuringiensis strain WS3 [61], involvement of hydroxyl, thiol, amide and amine functional groups for arsenic adsorption [62]. These studies reflect that arsenic resistant strains possess specific functional groups that facilitate arsenic adsorption on the cell surface limiting its entry into the cell. The arsenic sensitive strain NK11 (S6 Fig) dried bacterial biomass did not exhibit arsenic adsorption probably due to the absence of functional groups on the cell surface.

Thus, the isolated *Klebsiella oxytoca* environmental strain can remediate arsenic more efficiently than the previously reported *Bacillus cereus* which requires a biomass concentration of 6g/L for arsenic absorption. However, it is less efficient than *Pseudomonas aeruginosa*, which can efficiently remove 90% arsenic using 1g/L biomass. The results of this study demonstrated that the isolated *Klebsiella oxytoca* AT-02 strain has devised two different mechanisms to resist arsenic i.e. arsenite efflux and biofilm formation to restrict arsenate entry into the cell. Furthermore, its biomass can also be used for arsenic remediation.

## 5. Conclusion

Thus, from this study, it can be concluded that the isolated AT-02 strain can resist both arsenate and arsenite up to 10,000ppm and 600ppm respectively. Molecular analysis revealed activation of arsenic efflux pump to eject the toxic arsenite from the cell whereas biofilm formation was employed to evade arsenate. The strain was able to remediate arsenic within 30 minutes of incubation. FTIR data demonstrated the involvement of aromatic amino acids and the carbonyl groups in arsenic binding. Therefore, the arsenic resistance ability of the AT-02 strain can be exploited for bioremediation. However, future studies are required to determine the potential chemical groups to modify the bacterial biomass to enhance its bioremediation potential.

## 6 Declarations

### 6.1 Author contribution

Dr. Aamira Tariq has conceptualised, supervised the research and manuscript preparation. Mr.Ubaid and Ms.Hira were involved in the strain isolation, growth curves, bioremediation and FTIR analysis mentioned in Figs 1, 2 and 7. Ms.Sana waqar has conducted the experiments mentioned in the Fig 6 with respect to arsB expression and biofilm formation. Ms.Hadiqa Aimen was involved in the DNA extraction and sequencing of the strain and amplification of the arsB gene, phylogenetic analysis and homology modelling mentioned in Figs 3 and 4. Dr.Nazish and Dr.Sadia have critically reviewed and edited the manuscript.

## Supporting information

S1 Data (A). 16SrRNA amplification using different dilutions of DNA generating a PCR product of 1500b.p. (B) arsB amplification using gene primers yielding a product of 1293b.p.
(PDF)

S1 Fig Biochemical characterization of AT-02: **A.** Growth of arsenic resistant strain on nutrient agar plate with arsenic. **B.** Growth of AT-02 on MacConkey agar showing pink mucoid colonies followed by string test. **C.** Gram staining showing gram-negative rods, oxidase negative (no color change), catalase positive (bubble formation), TSI positive (gas production) followed by citrate positive test indicating the presence of *Klebsiella*. Indole test showing non-motile indole positive *Klebsiella*.
(TIF)

S2 Fig **A-D:** Stereochemical analysis based on PROCHECK showing Ramachandran plot based on ArsB three dimensional structures of *K*.*oxytoca*, *E*.*coli*, *K*.*pneumoniae* and *P*.*putida* showing 305, 309, 309 and 97 residues in the favoured region and 7, 4, 4,0 residues in the generously allowed region respectively indicating good quality of the predicted three dimensional structures. Among these four protein structures, three (*K*.*oxytoca*, *E*.*coli*, *K*.*pneumoniae)* had 1 residue (Lys133, Ile 290, Ile 290)in the disallowed region whereas *P*.*putida* ArsB had no residues in this region.
(TIF)

S3 Fig **A-D:** Errat is an empirical atom based program for validating protein structures. ArsB three dimensional structures of *K*.*oxytoca*, *E*.*coli*, *K*.*pneumoniae* and *P*.*putida* were evaluated. All models quality was assessed above 91% showing good quality of the predicted models.
(TIF)

S4 Fig **A-C:** Structural superimposition of ArsB of *K*.*oxytoca* with *E*.*coli*, *K*.*oxytoca* with *K*.*pneumoniae* and *K*.*oxytoca* with *P*.*putida* respectively with root mean square deviation (RMSD) value of 1.26, 1.26 and 2.06 respectively. “:” denotes aligned residue pairs. The conserved arsenic binding residues are highlighted in red boxes. Structural alignment showed that these conserved residues are perfectly aligned.
(TIF)

S5 Fig Determination of biofilm formation in the presence of arsenate: LB media with 1000ppm arsenate showed increased biofilm formation. (TIF)

S6 Fig Biosorption potential of arsenic sensitive *Klebsiella pneumonaie* strain (NK11): A. Incubation of NK11 bacterial biomass with 100ppb working solution showed reduced adsorption of arsenic at 0.5hr (98ppb), 1hr(95.6ppb) and 2hr(94.2ppb). B. Arsenic remediation efficiency of NK11 biomass (0.5hr 1.6%, 1hr 4.3% and 2hr 5.7% respectively) calculated at different time points.
(TIF)

S1 File AT-02 resistance against different concentrations of arsenate. (XLSX)

S2 File AT-02 resistance against different concentrations of arsenite. (XLSX)

S3 File K p resistance against different concentrations of arsenate. (XLSX)

S4 File Kp resistance against different concentrations of arsenite. (XLSX)

S5 File Comparison of Kp and Ko at different arsenate concentration. (XLSX)

S6 File Comparison of Kp and Ko at different arsenite concentration. (XLSX)

S7 File Arsenic remediation by AT-02 biomass. (XLSX)

S8 File Remediation efficiency of AT-02 biomass. (XLSX)

S9 File Arsenic remediation by Kp(NK11) biomass. (XLSX)

S10 File Remediation efficiency of Kp(NK11) biomass. (XLSX)

S1 Table Physio-chemical properties of the water from Multan, Pakistan. (DOCX)

S2 Table Primers used for expression arsB expression analysis. (DOCX)

S3 Table Arsb arsenic binding residues mutation consequence prediction by PROVEAN. (DOCX)

S4 Table Arsb arsenic binding residues mutation consequence prediction by DDMUT on protein stability. (DOCX)
