# Supplementary material for: Arsenic efflux and bioremediation potential of Klebsiella oxytoca via the arsB gene
Source: PLoS One. 2025 Jan 29;20(1):e0307918. doi: 10.1371/journal.pone.0307918 (PMC11778763; doi:10.1371/journal.pone.0307918)
Supplement: S1 Data — (A). 16SrRNA amplification using different dilutions of DNA generating a PCR product of 1500b.p. (B) arsB amplification using gene primers yielding a product of 1293b.p. (PDF) [file pone.0307918.s001.pdf]

S1 raw

16S PCR

A

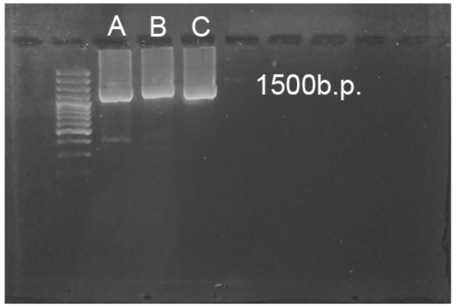

arsB amplification

B

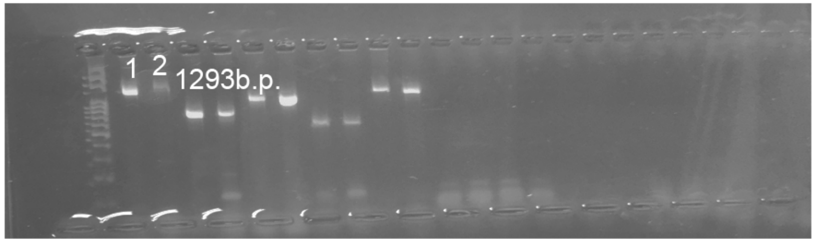

Table T1

AT-02 resistance against different concentrations of arsenate

Arsenate Resistant Bacteria in plain LB Broth																															
																	Arsenate														
Time		O.D at 600nm																													
		LB + Bacteria					1000ppm					3000ppm					5000ppm					7000ppm					10000ppm				
0		0.2922	0.296	0.286	0.2946	0.005414795	0.29	0.29	0.3	0.28	0.01	0.288666667	0.286	0.29	0.29	0.002309401	0.295333333	0.296	0.29	0.3	0.005033223	0.291266667	0.2938	0.29	0.29	0.002193931	0.299333333	0.29	0.298	0.31	0.010
2		0.4495	0.435	0.45	0.4635	0.014256577	0.357333333	0.362	0.36	0.35	0.006429101	0.362666667	0.339	0.389	0.36	0.025106441	0.325033333	0.341	0.31	0.3241	0.015521061	0.318033333	0.321	0.3161	0.317	0.00260832	0.305766667	0.2973	0.3	0.32	0.012
4		0.5268	0.54	0.524	0.5164	0.012046576	0.412666667	0.41	0.408	0.42	0.006429101	0.3845	0.3885	0.395	0.37	0.012971122	0.3619	0.3657	0.37	0.35	0.010527583	0.3443	0.3349	0.349	0.349	0.008140639	0.3151	0.3053	0.31	0.33	0.013
6		0.621333333	0.624	0.64	0.6	0.020132892	0.471	0.48	0.47	0.463	0.008544004	0.464866667	0.4446	0.46	0.49	0.023087948	0.426666667	0.455	0.425	0.4	0.027537853	0.348066667	0.3472	0.348	0.349	0.00090185	0.330133333	0.3264	0.324	0.34	0.008
8		0.7154	0.72	0.71	0.7162	0.005047772	0.541	0.543	0.53	0.55	0.010148892	0.533033333	0.5191	0.53	0.55	0.015671737	0.49	0.49	0.483	0.497	0.007	0.360433333	0.3583	0.36	0.363	0.002379776	0.353266667	0.3498	0.35	0.36	0.005
10		0.762733333	0.7642	0.774	0.75	0.012067035	0.600366667	0.5911	0.6	0.61	0.009455334	0.5729	0.5687	0.59	0.56	0.015434701	0.554566667	0.5367	0.557	0.57	0.016782829	0.403366667	0.3991	0.401	0.41	0.005822657	0.377033333	0.3791	0.371	0.381	0.005
12		0.781333333	0.787	0.767	0.79	0.012503333	0.6546	0.648	0.6558	0.66	0.006089335	0.639433333	0.6383	0.61	0.67	0.030016051	0.622366667	0.6151	0.621	0.631	0.00803762	0.4486	0.4398	0.448	0.458	0.009114823	0.398466667	0.3882	0.3982	0.409	0.010
14		0.82	0.82	0.8	0.84	0.02	0.716266667	0.7	0.7188	0.73	0.015159595	0.6725	0.6975	0.65	0.67	0.023848848	0.663066667	0.6552	0.662	0.672	0.008450641	0.531433333	0.5213	0.53	0.543	0.010920775	0.419433333	0.3983	0.43	0.43	0.018
16		0.879333333	0.868	0.88	0.89	0.011015141	0.782366667	0.771	0.7851	0.791	0.010276348	0.768633333	0.7579	0.769	0.779	0.010554778	0.7299	0.7197	0.73	0.74	0.010150369	0.6212	0.6016	0.626	0.636	0.017695197	0.4471	0.4213	0.45	0.47	0.024
18		0.937333333	0.952	0.94	0.92	0.016165808	0.869033333	0.8761	0.85	0.881	0.016664433	0.852866667	0.8486	0.86	0.85	0.00621718	0.813966667	0.8119	0.83	0.8	0.0151064	0.655166667	0.6455	0.655	0.665	0.009751068	0.4597	0.4371	0.461	0.481	0.021
20		0.967	0.942	0.962	0.972	0.015275252	0.826466667	0.8414	0.834	0.804	0.019805386	0.812833333	0.8235	0.8	0.815	0.011898879	0.808633333	0.8039	0.813	0.809	0.004561067	0.6279	0.6267	0.627	0.63	0.001824829	0.439933333	0.4138	0.438	0.468	0.027
22		0.91825	0.9	0.9165	0.92	0.01068098	0.7777	0.7931	0.78	0.76	0.016669433	0.76	0.75	0.76	0.77	0.01	0.7457	0.7451	0.741	0.751	0.005026927	0.615233333	0.6087	0.617	0.62	0.005853489	0.4292	0.4116	0.426	0.45	0.019
24		0.864	0.89	0.869	0.859	0.015821926	0.727366667	0.7411	0.711	0.73	0.015221805	0.7213	0.7289	0.73	0.71	0.010250366	0.7166	0.7198	0.7	0.73	0.015253852	0.5949	0.58	0.59	0.599	0.009504385	0.4102	0.3946	0.4	0.436	0.022

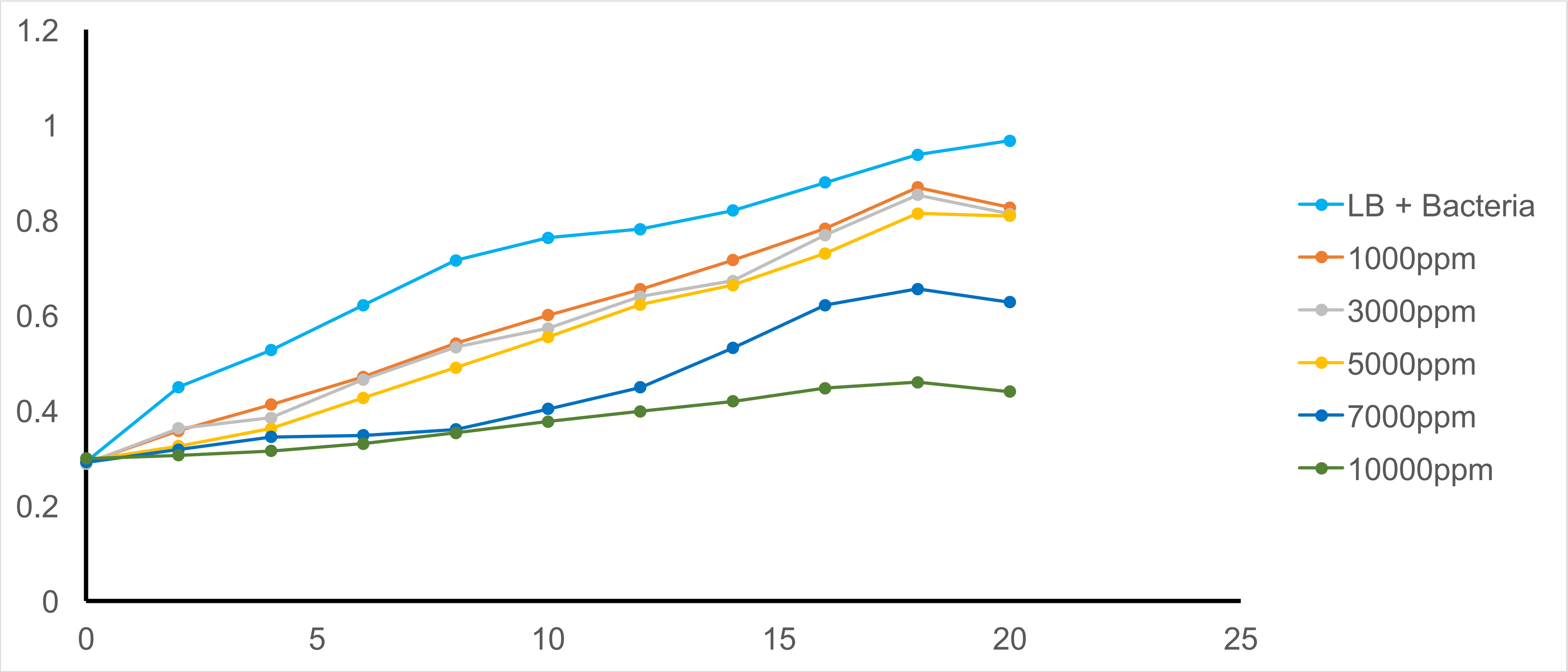

1.2
1
0.8
0.6
0.4
0.2
0
0
5
10
15
20
25
LB + Bacteria
1000ppm
3000ppm
5000ppm
7000ppm
10000ppm

Table T2

AT-02 resistance against different concentrations of arsenite

Time	O.D at 600nm																											
	LB + Bacteria					200					400					600					800					200ppm	400ppm	600ppm	800ppm
0	0.295666667	0.296	0.295	0.296	0.00057735	0.2935	0.2945	0.295	0.291	0.0021794	0.2887	0.289	0.298	0.279	0.009504385	0.2926	0.2987	0.29	0.289	0.005335104	0.288	0.287	0.29	0.287	0.0017	0.2098	0.1492	0.12514	4.9423E-06
2	0.460666667	0.465	0.467	0.45	0.009291573	0.3751	0.374	0.376	0.3754	0.0010263	0.3304	0.3342	0.347	0.31	0.018790423	0.3227	0.3242	0.342	0.302	0.020040293	0.288	0.287	0.29	0.287	0.0017				
4	0.516733333	0.5164	0.5274	0.5064	0.010503968	0.4158	0.428	0.4195	0.4	0.0143556	0.374	0.376	0.386	0.36	0.013114877	0.3709	0.3706	0.386	0.356	0.015001778	0.288	0.287	0.29	0.287	0.0017				
6	0.622666667	0.624	0.64	0.604	0.018036999	0.4696	0.47	0.4517	0.487	0.017654	0.4433	0.44	0.46	0.43	0.015275252	0.4379	0.4387	0.457	0.418	0.019512304	0.3	0.29	0.31	0.3	0.01				
8	0.714133333	0.7162	0.72	0.7062	0.007128347	0.5372	0.527	0.5327	0.552	0.013102	0.5288	0.5272	0.542	0.5172	0.012477179	0.5171	0.532	0.502	0.5172	0.015000444	0.3	0.29	0.31	0.3	0.01				
10	0.762733333	0.7642	0.782	0.742	0.020040293	0.5999	0.597	0.5827	0.62	0.0188183	0.5783	0.5769	0.589	0.569	0.010073232	0.5653	0.5669	0.58	0.549	0.015561812	0.3103	0.3	0.321	0.31	0.0105				
12	0.7939	0.7877	0.817	0.777	0.020708211	0.6585	0.66	0.6428	0.6728	0.0150537	0.6333	0.63	0.65	0.62	0.015275252	0.6269	0.6267	0.637	0.617	0.0100015	0.3103	0.3	0.321	0.31	0.0105				
14	0.810733333	0.8112	0.82	0.801	0.009508593	0.7173	0.7035	0.7135	0.735	0.0160961	0.694	0.692	0.71	0.68	0.015099669	0.6827	0.698	0.662	0.6882	0.018612182	0.34	0.33	0.35	0.34	0.01				
16	0.867866667	0.8668	0.88	0.8568	0.011636723	0.7541	0.756	0.7642	0.742	0.0112256	0.7555	0.7524	0.774	0.74	0.0172062	0.7491	0.7424	0.7624	0.7424	0.011547005	0.34	0.33	0.35	0.34	0.01				
18	0.9374	0.9372	0.943	0.932	0.005502727	0.8671	0.864	0.884	0.8534	0.0155388	0.8499	0.8438	0.868	0.838	0.015912678	0.8352	0.8338	0.858	0.8138	0.022133233	0.34	0.33	0.35	0.34	0.01				
20	0.9524	0.9532	0.972	0.932	0.020011996	0.8321	0.8324	0.84	0.824	0.0080033	0.8291	0.8291	0.8491	0.8091	0.02	0.821	0.8211	0.841	0.801	0.020000083	0.34	0.33	0.35	0.34	0.01				
22	0.918833333	0.9165	0.935	0.905	0.015135499	0.7821	0.7821	0.7821	0.7821	1.36E-16	0.7733	0.775	0.795	0.75	0.022546249	0.7542	0.7675	0.75	0.745	0.011814539	0.3	0.29	0.31	0.3	0.01				
24	0.872333333	0.869	0.889	0.859	0.015275252	0.7321	0.7321	0.7321	0.7321	0	0.726	0.726	0.74	0.706	0.017088007	0.7162	0.7216	0.726	0.701	0.01334616	0.28	0.27	0.29	0.28	0.01				

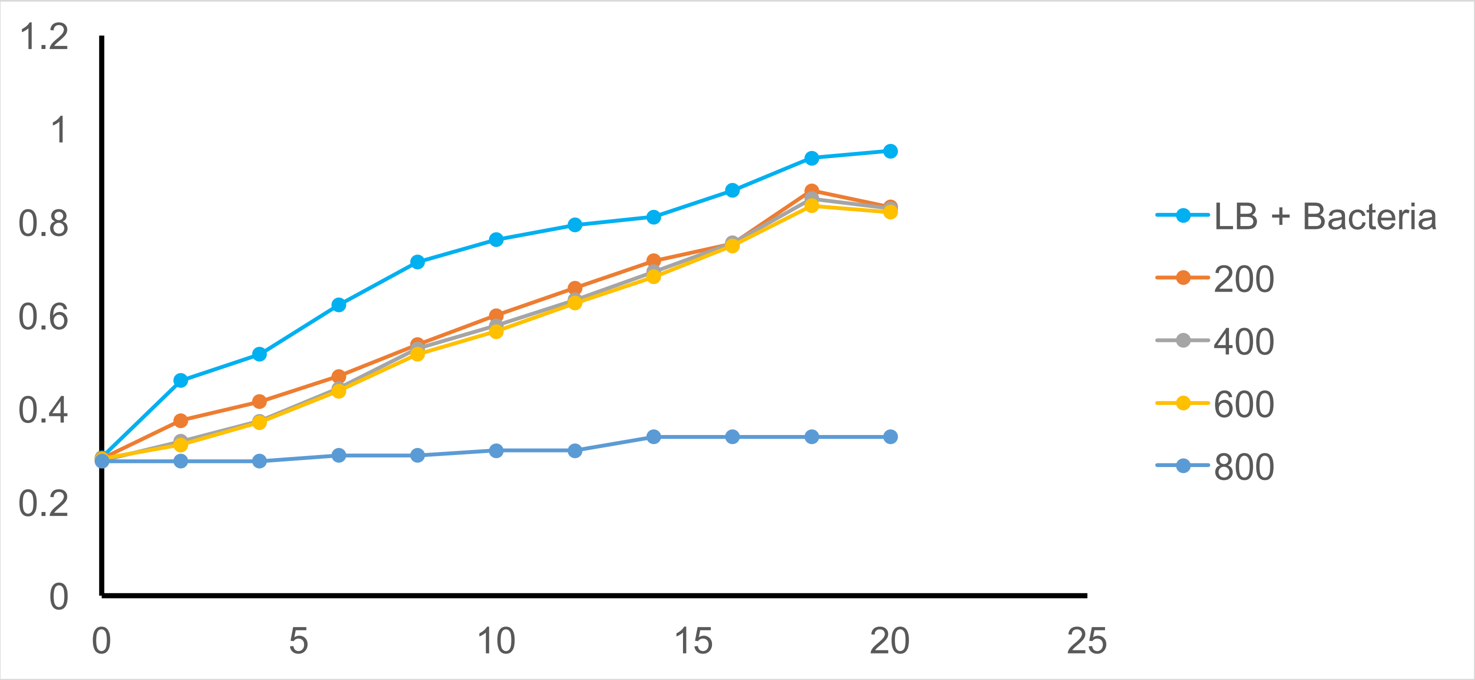

Table T3

Controlled Klebsiella Pneumoniae Arsenate Concentration Gowth Curve

Time	O.D at 600nm									
	LB + Bacteria					1000ppm				
0	0.2922	0.2946	0.286	0.296	0.0054	0.294	0.295	0.288	0.299	0.0056
2	0.485	0.47	0.485	0.5	0.015	0.311333333	0.312	0.32	0.302	0.009
4	0.574666667	0.55	0.58	0.594	0.0225	0.327333333	0.341	0.331	0.31	0.0158
6	0.713333333	0.68	0.72	0.74	0.0306	0.341333333	0.35	0.354	0.32	0.0186
8	0.777733333	0.762	0.782	0.7892	0.0141	0.354333333	0.36	0.363	0.34	0.0125
10	0.851333333	0.842	0.85	0.862	0.0101	0.371	0.371	0.381	0.361	0.01
12	0.937233333	0.9277	0.937	0.947	0.0097	0.415666667	0.41	0.435	0.402	0.0172
14	1.124	1.102	1.12	1.15	0.0242	0.439333333	0.44	0.453	0.425	0.014
16	1.278666667	1.268	1.28	1.288	0.0101	0.461	0.461	0.471	0.451	0.01
18	1.381333333	1.372	1.38	1.392	0.0101	0.480666667	0.482	0.49	0.47	0.0101
20	1.462066667	1.4562	1.462	1.468	0.0059	0.513333333	0.513	0.524	0.503	0.0105
22	1.136666667	1.065	1.165	1.18	0.0625	0.474333333	0.47	0.49	0.463	0.014
24	0.982666667	0.969	0.989	0.99	0.0118	0.423	0.41	0.451	0.408	0.0243

Time										
	3000ppm					5000ppm				
0	0.293186667	0.2976	0.286	0.296	0.0063	0.291733333	0.2936	0.296	0.2856	0.0054
2	0.303333333	0.31	0.3	0.3	0.0058	0.297	0.301	0.301	0.289	0.0069
4	0.316666667	0.33	0.32	0.3	0.0153	0.306666667	0.31	0.31	0.3	0.0058
6	0.327666667	0.34	0.33	0.313	0.0137	0.321666667	0.325	0.325	0.315	0.0058
8	0.334666667	0.345	0.339	0.32	0.0131	0.327666667	0.33	0.33	0.323	0.004
10	0.354333333	0.358	0.36	0.345	0.0081	0.342	0.34	0.346	0.34	0.0035
12	0.373666667	0.375	0.386	0.36	0.0131	0.357333333	0.35	0.357	0.365	0.0075
14	0.39	0.39	0.4	0.38	0.01	0.368666667	0.372	0.362	0.372	0.0058
16	0.417	0.419	0.429	0.403	0.0131	0.406	0.409	0.39	0.419	0.0147
18	0.440666667	0.446	0.45	0.426	0.0129	0.427033333	0.421	0.431	0.4291	0.0053
20	0.469	0.48	0.47	0.457	0.0115	0.435666667	0.43	0.44	0.437	0.0051
22	0.41	0.41	0.42	0.4	0.01	0.396666667	0.39	0.4	0.4	0.0058
24	0.383	0.399	0.38	0.37	0.0147	0.366666667	0.35	0.37	0.38	0.0153

Time											
	7000ppm					10000ppm					
0	0.2936	0.2938	0.298	0.289	0.0045	0.2914	0.2871	0.29	0.2971	0.0051	
2	0.295366667	0.2961	0.299	0.291	0.0041	0.294333333	0.29	0.293	0.3	0.0051	
4	0.299333333	0.299	0.3	0.299	0.0006	0.303	0.293	0.296	0.32	0.0148	
6	0.312066667	0.3072	0.312	0.317	0.0049	0.314666667	0.3	0.304	0.34	0.022	
8	0.319	0.313	0.32	0.324	0.0056	0.322666667	0.308	0.31	0.35	0.0237	
10	0.326666667	0.321	0.329	0.33	0.0049	0.336033333	0.31	0.319	0.3791	0.0376	
12	0.336333333	0.33	0.338	0.341	0.0057	0.343	0.32	0.327	0.382	0.034	
14	0.363333333	0.35	0.36	0.38	0.0153	0.354333333	0.33	0.34	0.393	0.0339	
16	0.394333333	0.38	0.39	0.413	0.0169	0.369	0.34	0.35	0.417	0.0419	
18	0.413333333	0.405	0.415	0.42	0.0076	0.384333333	0.361	0.371	0.421	0.0321	
20	0.42	0.41	0.42	0.43	0.01	0.403333333	0.38	0.39	0.44	0.0321	
22	0.376666667	0.36	0.38	0.39	0.0153	0.329333333	0.316	0.326	0.346	0.0153	
24	0.321	0.309	0.329	0.325	0.0106	0.2822	0.2746	0.286	0.286	0.0066	

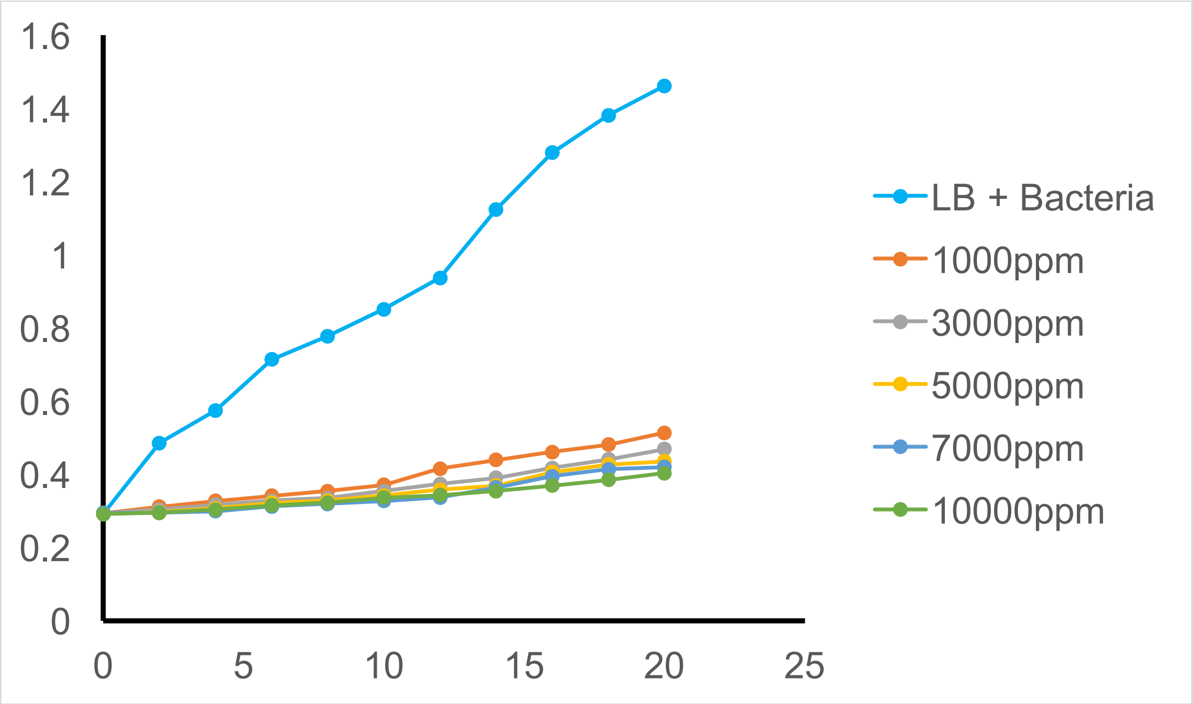
1.6
1.4
1.2
1
0.8
0.6
0.4
0.2
0
0
5
10
15
20
25
LB + Bacteria
1000ppm
3000ppm
5000ppm
7000ppm
10000ppm

Table T4

Controlled Klebsiella Pneumoniae Arsenite Concentration Gowth

Time	O.D at 600nm									
	LB + Bacteria	200ppm								
0	0.294866667	0.29								
2	0.482666667	0.3133								
4	0.541333333	0.3303								
6	0.671333333	0.35								
8	0.741333333	0.37								
10	0.821333333	0.39								
12	0.908233333	0.4								
14	1.017333333	0.429								
16	1.133466667	0.4593								
18	1.348	0.4797								
20	1.435066667	0.4933								
22	1.145	0.4033								
24	0.9623	0.3117								
Time	O.D at 600nm									
	LB + Bacteria					200ppm				
0	0.294866667	0.2946	0.296	0.294	0.001	0.29	0.285	0.29	0.295	0.005
2	0.482666667	0.493	0.485	0.47	0.0117	0.3133	0.3	0.31	0.33	0.0153
4	0.541333333	0.564	0.54	0.52	0.022	0.3303	0.321	0.33	0.34	0.0095
6	0.671333333	0.694	0.67	0.65	0.022	0.35	0.34	0.35	0.36	0.01
8	0.741333333	0.762	0.742	0.72	0.021	0.37	0.36	0.37	0.38	0.01
10	0.821333333	0.842	0.82	0.802	0.02	0.39	0.38	0.39	0.4	0.01
12	0.908233333	0.9277	0.907	0.89	0.0189	0.4	0.39	0.4	0.41	0.01
14	1.017333333	1.112	1.02	0.92	0.096	0.429	0.41	0.437	0.44	0.0165
16	1.133466667	1.2668	1.0668	1.0668	0.1155	0.4593	0.43	0.468	0.48	0.0261
18	1.348	1.372	1.352	1.32	0.0262	0.4797	0.45	0.49	0.499	0.0261
20	1.435066667	1.4532	1.432	1.42	0.0168	0.4933	0.46	0.5	0.52	0.0306
22	1.145	1.165	1.14	1.13	0.018	0.4033	0.39	0.4	0.42	0.0153
24	0.9623	0.9869	0.96	0.94	0.0235	0.3117	0.29	0.3	0.345	0.0293

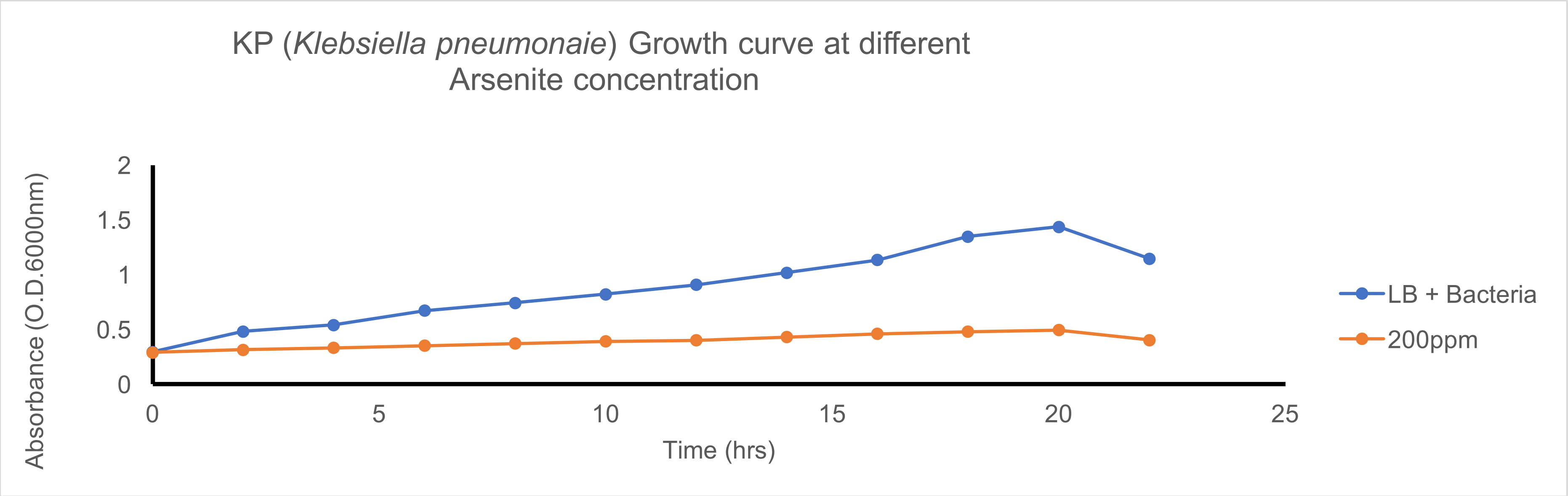
KP (*Klebsiella pneumonaie*) Growth curve at different
Arsenite concentration
Absorbance (O.D.6000nm)
2
1.5
1
0.5
0
0
5
10
15
20
25
Time (hrs)
LB + Bacteria
200ppm

Table T5

Comparison of Kp and Ko

Kp arsenate

O.D at 600nm																													
LB + Bacteria					1000ppm					3000ppm					5000ppm					7000ppm					10000ppm				
**0.2922**	**0.2946**	**0.286**	**0.296**	0.005414795	**0.294**	0.295	**0.288**	0.299	0.005568	**0.293186667**	0.29756	0.286	0.296	0.006273	**0.291733333**	0.2936	0.296	0.2856	0.005445	**0.2936**	0.2938	0.298	0.289	0.004503	**0.2914**	0.2871	0.29	0.2971	0.005145
**0.485**	**0.47**	**0.485**	**0.5**	0.015	**0.311333333**	0.312	0.32	0.302	0.009018	**0.303333333**	0.31	0.3	0.3	0.005774	**0.297**	0.301	0.301	0.289	0.006928	**0.295366667**	0.2961	0.299	0.291	0.00405	**0.294333333**	0.29	0.293	0.3	0.005132
**0.574666667**	**0.55**	**0.58**	**0.594**	0.02247962	**0.327333333**	0.341	0.331	0.31	0.015822	**0.316666667**	0.33	0.32	0.3	0.015275	**0.306666667**	0.31	0.31	0.3	0.005774	**0.299333333**	0.299	0.3	0.299	0.000577	**0.299666667**	0.293	0.296	0.31	0.009074
**0.713333333**	**0.68**	**0.72**	**0.74**	0.030550505	**0.341333333**	0.35	0.354	0.32	0.018583	**0.327666667**	0.34	0.33	0.313	0.01365	**0.321666667**	0.325	0.325	0.315	0.005774	**0.312066667**	0.3072	0.312	0.317	0.0049	**0.306**	0.3	0.304	0.314	0.007211
**0.777733333**	**0.762**	**0.782**	**0.7892**	0.014093024	**0.354333333**	0.36	0.363	0.34	0.012503	**0.334666667**	0.345	0.339	0.32	0.013051	**0.327666667**	0.33	0.33	0.323	0.004041	**0.319**	0.313	0.32	0.324	0.005568	**0.312666667**	0.308	0.31	0.32	0.006429
**0.851333333**	**0.842**	**0.85**	**0.862**	0.010066446	**0.371**	0.371	0.381	0.361	0.01	**0.354333333**	0.358	0.36	0.345	0.008145	**0.342**	0.34	0.346	0.34	0.003464	**0.326666667**	0.321	0.329	0.33	0.004933	**0.319666667**	0.31	0.319	0.33	0.010017
**0.937233333**	**0.9277**	**0.937**	**0.947**	0.009652115	**0.415666667**	0.41	0.435	0.402	0.017214	**0.373666667**	0.375	0.386	0.36	0.013051	**0.357333333**	0.35	0.357	0.365	0.007506	**0.336333333**	0.33	0.338	0.341	0.005686	**0.329**	0.32	0.327	0.34	0.010149
**1.124**	**1.102**	**1.12**	**1.15**	0.024248711	**0.439333333**	0.44	0.453	0.425	0.014012	**0.39**	0.39	0.4	0.38	0.01	**0.368666667**	0.372	0.362	0.372	0.005774	**0.363333333**	0.35	0.36	0.38	0.015275	**0.341**	0.33	0.34	0.353	0.011533
**1.278666667**	**1.268**	**1.28**	**1.288**	0.010066446	**0.461**	0.461	0.471	0.451	0.01	**0.417**	0.419	0.429	0.403	0.013115	**0.406**	0.409	0.39	0.419	0.014731	**0.394333333**	0.38	0.39	0.413	0.016921	**0.35**	0.34	0.35	0.36	0.01
**1.381333333**	**1.372**	**1.38**	**1.392**	0.010066446	**0.480666667**	0.482	0.49	0.47	0.010066	**0.440666667**	0.446	0.45	0.426	0.012858	**0.427033333**	0.421	0.431	0.4291	0.005311	**0.413333333**	0.405	0.415	0.42	0.007638	**0.371**	0.361	0.371	0.381	0.01
**1.462066667**	**1.4562**	**1.462**	**1.468**	0.005900282	**0.513333333**	0.513	0.524	0.503	0.010504	**0.469**	0.48	0.47	0.457	0.011533	**0.435666667**	0.43	0.44	0.437	0.005132	**0.42**	0.41	0.42	0.43	0.01	**0.39**	0.38	0.39	0.4	0.01
**1.136666667**	**1.065**	**1.165**	**1.18**	0.062516664	**0.474333333**	0.47	0.49	0.463	0.014012	**0.41**	0.41	0.42	0.4	0.01	**0.396666667**	0.39	0.4	0.4	0.005774	**0.376666667**	0.36	0.38	0.39	0.015275	**0.324**	0.316	0.326	0.33	0.007211
**0.982666667**	0.969	0.989	0.99	0.011846237	**0.423**	0.41	0.451	0.408	0.024269	**0.383**	0.399	0.38	0.37	0.014731	**0.366666667**	0.35	0.37	0.38	0.015275	**0.321**	0.309	0.329	0.325	0.010583	**0.285533333**	0.2746	0.286	0.296	0.010708

AT-02 as Ko

Time	O.D at 600nm																													
	LB + Bacteria					1000ppm					3000ppm					5000ppm					7000ppm					10000ppm				
0	**0.2922**	**0.296**	**0.286**	**0.2946**	**0.005414795**	0.29	0.29	0.3	0.28	**0.01**	0.288666667	0.286	0.29	0.29	**0.002309401**	0.295333333	0.296	0.29	0.3	**0.005033223**	0.291266667	0.2938	0.29	0.29	**0.002193931**	0.299333333	0.29	0.298	0.31	**0.010066446**
2	**0.4495**	**0.435**	**0.45**	**0.4635**	**0.014256577**	0.357333333	0.362	0.36	0.35	**0.006429101**	0.362666667	0.339	0.389	0.36	**0.02510644**	0.325033333	0.341	0.31	0.3241	**0.015521061**	0.318033333	0.321	0.3161	0.317	**0.00260832**	0.305766667	0.2973	0.3	0.32	**0.012400134**
4	**0.5268**	**0.54**	**0.524**	**0.5164**	**0.012046576**	0.412666667	0.41	0.408	0.42	**0.006429101**	0.3845	0.3885	0.395	0.37	**0.012971122**	0.3619	0.3657	0.37	0.35	**0.010527583**	0.3443	0.3349	0.349	0.349	**0.008140639**	0.3151	0.3053	0.31	0.33	**0.013116021**
6	**0.621333333**	**0.624**	**0.64**	**0.6**	**0.020132892**	0.471	0.48	0.47	0.463	**0.008544004**	0.464866667	0.4446	0.46	0.49	**0.023087948**	0.426666667	0.455	0.425	0.4	**0.027537853**	0.348066667	0.3472	0.348	0.349	**0.00090185**	0.330133333	0.3264	0.324	0.34	**0.008628634**
8	**0.7154**	**0.72**	**0.71**	**0.7162**	**0.005047772**	0.541	0.543	0.53	0.55	**0.010148892**	0.533033333	0.5191	0.53	0.55	**0.015671737**	0.49	0.49	0.483	0.497	**0.007**	0.360433333	0.3583	0.36	0.363	**0.002379776**	0.353266667	0.3498	0.35	0.36	**0.005832095**
10	**0.762733333**	**0.7642**	**0.774**	**0.75**	**0.012067035**	0.600366667	0.5911	0.6	0.61	**0.009455334**	0.5729	0.5687	0.59	0.56	**0.015434701**	0.554566667	0.5367	0.557	0.57	**0.016782829**	0.403366667	0.3991	0.401	0.41	**0.005822657**	0.377033333	0.3791	0.371	0.381	**0.005310681**
12	**0.781333333**	**0.787**	**0.767**	**0.79**	**0.012503333**	0.6546	0.648	0.6558	0.66	**0.006089335**	0.639433333	0.6383	0.61	0.67	**0.030016051**	0.622366667	0.6151	0.621	0.631	**0.00803762**	0.4486	0.4398	0.448	0.458	**0.009114823**	0.398466667	0.3882	0.3982	0.409	**0.010402564**
14	**0.82**	**0.82**	**0.8**	**0.84**	**0.02**	0.716266667	0.7	0.7188	0.73	**0.015159595**	0.6725	0.6975	0.65	0.67	**0.02384848**	0.663066667	0.6552	0.662	0.672	**0.008450641**	0.531433333	0.5213	0.53	0.543	**0.010920775**	0.419433333	0.3983	0.43	0.43	**0.018302004**
16	**0.879333333**	**0.868**	**0.88**	**0.89**	**0.011015141**	0.782366667	0.771	0.7851	0.791	**0.010276348**	0.768633333	0.7579	0.769	0.779	**0.010554778**	0.7299	0.7197	0.73	0.74	**0.010150369**	0.6212	0.6016	0.626	0.636	**0.017695197**	0.4471	0.4213	0.45	0.47	**0.024479175**
18	**0.937333333**	**0.952**	**0.94**	**0.92**	**0.016165808**	0.869033333	0.8761	0.85	0.881	**0.016664433**	0.852866667	0.8486	0.86	0.85	**0.00621718**	0.813966667	0.8119	0.83	0.8	**0.0151064**	0.655166667	0.6455	0.655	0.665	**0.009751068**	0.4597	0.4371	0.461	0.481	**0.021978853**
20	**0.967**	**0.942**	**0.962**	**0.972**	**0.015275252**	0.826466667	0.8414	0.834	0.804	**0.019805386**	0.812833333	0.8235	0.8	0.815	**0.011898879**	0.808633333	0.8039	0.813	0.809	**0.004561067**	0.6279	0.6267	0.627	0.63	**0.001824829**	0.439933333	0.4138	0.438	0.468	**0.027151673**
22	**0.91825**	**0.9**	**0.9165**	**0.92**	**0.01068098**	0.7777	0.7931	0.78	0.76	**0.016669433**	0.76	0.75	0.76	0.77	**0.01**	0.7457	0.7451	0.741	0.751	**0.005026927**	0.615233333	0.6087	0.617	0.62	**0.005853489**	0.4292	0.4116	0.426	0.45	**0.019398969**
24	**0.864**	0.89	0.869	0.859	**0.015821926**	0.727366667	0.7411	0.711	0.73	**0.015221805**	0.7213	0.7239	0.73	0.71	**0.010250366**	0.7166	0.7198	0.7	0.73	**0.015253852**	0.5949	0.58	0.59	0.599	**0.009504385**	0.4102	0.3946	0.4	0.436	**0.022505999**

Comparison of Kp and Ko at different arsenate concentration

	KP LB	AT-02 LB	KP 1000ppm	AT-02 1000ppm	KP 3000ppm	AT-02 3000ppm	KP 5000ppm	AT-02 5000ppm	KP 7000ppm	AT-02 7000ppm	KP 10000ppm	AT-02 10000ppm	LB	1000ppm	3000ppm	5000ppm	7000ppm	10000ppm
0	0.2922	0.2922	**0.294**	0.29	**0.293186667**	0.288666667	**0.291733333**	0.295333333	**0.2936**	0.291266667	**0.2914**	0.299333333	0.156400864	0.00463455	0.001915362	0.002799637	0.022699788	0.00318
2	0.485	0.4495	**0.311333333**	0.357333333	**0.296666667**	0.362666667	**0.297**	0.325033333	**0.295366667**	0.318033333	**0.294333333**	0.305766667						
4	0.574666667	0.5268	**0.327333333**	0.412666667	**0.303333333**	0.3845	**0.306666667**	0.3619	**0.299333333**	0.3443	**0.299666667**	0.3151						
6	0.713333333	0.621333333	**0.341333333**	0.471	**0.314333333**	0.464866667	**0.321666667**	0.426666667	**0.312066667**	0.348066667	**0.306**	0.330133333						
8	0.777733333	0.7154	**0.354333333**	0.541	**0.334666667**	0.533033333	**0.327666667**	0.49	**0.319**	0.360433333	**0.312666667**	0.353266667						
10	0.851333333	0.762733333	**0.371**	0.600366667	**0.354333333**	0.5729	**0.342**	0.554566667	**0.326666667**	0.403366667	**0.319666667**	0.377033333						
12	0.937233333	0.781333333	**0.415666667**	0.6546	**0.373666667**	0.639433333	**0.357333333**	0.622366667	**0.336333333**	0.4486	**0.329**	0.398466667						
14	1.124	0.82	**0.439333333**	0.716266667	**0.39**	0.6725	**0.368666667**	0.663066667	**0.363333333**	0.531433333	**0.341**	0.419433333						
16	1.278666667	0.879333333	**0.461**	0.782366667	**0.417**	0.768633333	**0.406**	0.7299	**0.394333333**	0.6212	**0.35**	0.4471						
18	1.381333333	0.937333333	**0.480666667**	0.869033333	**0.440666667**	0.852866667	**0.427033333**	0.813966667	**0.413333333**	0.655166667	**0.371**	0.4597						
20	1.462066667	0.967	**0.513333333**	0.826466667	**0.469**	0.812833333	**0.435666667**	0.808633333	**0.42**	0.6279	**0.39**	0.439933333						
22	1.136666667	0.91825	**0.474333333**	0.7777	**0.41**	0.76	**0.396666667**	0.7457	**0.376666667**	0.615233333	**0.324**	0.4292						
24	0.982666667	0.864	**0.423**	0.727366667	**0.383**	0.7213	**0.366666667**	0.7166	**0.321**	0.5949	**0.285533333**	0.4102						

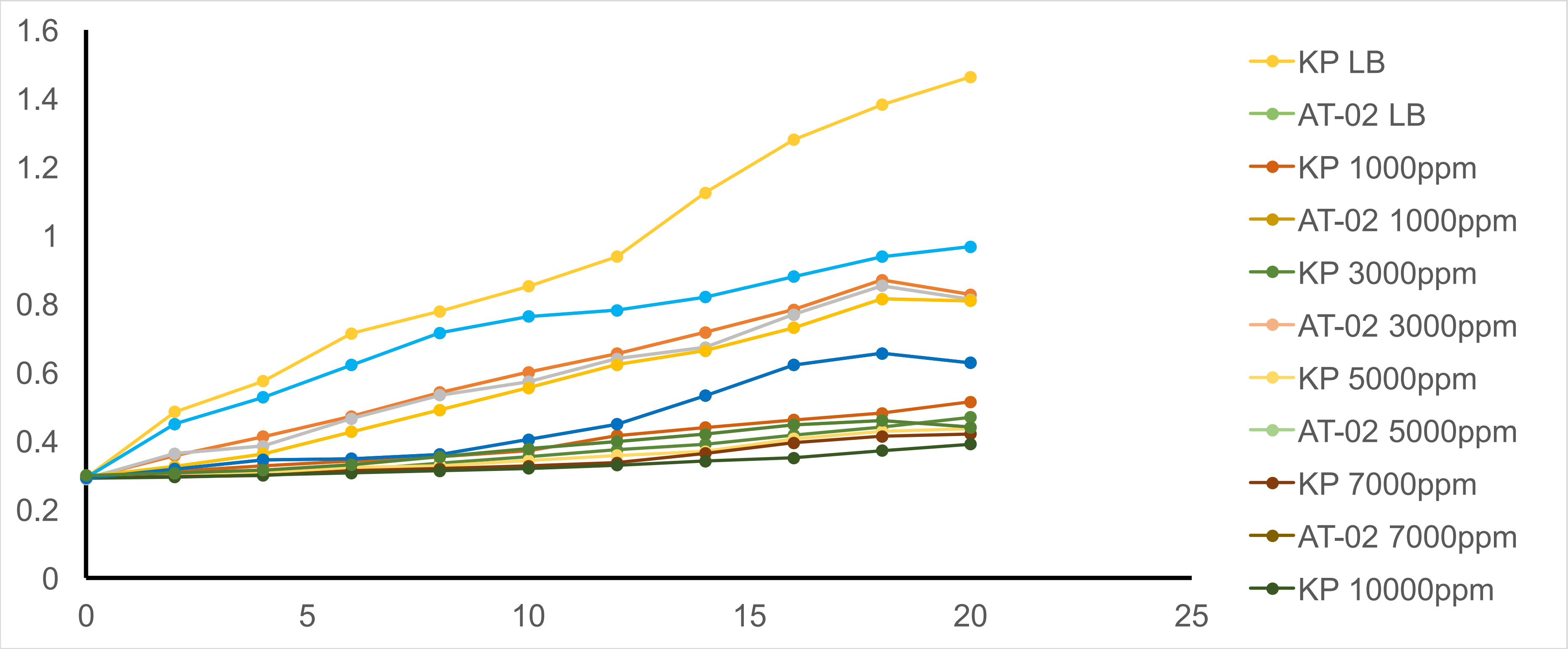
1.6
1.4
1.2
1
0.8
0.6
0.4
0.2
0
0
5
10
15
20
25
KP LB
AT-02 LB
KP 1000ppm
AT-02 1000ppm
KP 3000ppm
AT-02 3000ppm
KP 5000ppm
AT-02 5000ppm
KP 7000ppm
AT-02 7000ppm
KP 10000ppm

Table T6

Comparison of Kpand Ko at different arsenite concentration

AT-02

AT-02											
	**Time**	**O.D at 600nm**									
		**LB + Bacteria**					**200**				
	**0**	**0.295666667**	**0.296**	**0.295**	**0.296**	**0.00057735**	0.2935	0.2945	0.295	0.291	**0.002179449**
	**2**	**0.460666667**	**0.465**	**0.467**	**0.45**	**0.009291573**	0.375133333	0.374	0.376	0.3754	**0.00102632**
	**4**	**0.516733333**	**0.5164**	**0.5274**	**0.5064**	**0.010503968**	0.415833333	0.428	0.4195	0.4	**0.014355603**
	**6**	**0.622666667**	**0.624**	**0.64**	**0.604**	**0.018036999**	0.469566667	0.47	0.4517	0.487	**0.017653989**
	**8**	**0.714133333**	**0.7162**	**0.72**	**0.7062**	**0.007128347**	0.537233333	0.527	0.5327	0.552	**0.013102035**
	**10**	**0.762733333**	**0.7642**	**0.782**	**0.742**	**0.020040293**	0.5999	0.597	0.5827	0.62	**0.018818342**
	**12**	**0.7939**	**0.7877**	**0.817**	**0.777**	**0.020708211**	0.658533333	0.66	0.6428	0.6728	**0.015053682**
	**14**	**0.810733333**	**0.8112**	**0.82**	**0.801**	**0.009508593**	0.717333333	0.7085	0.7135	0.735	**0.016096066**
	**16**	**0.867866667**	**0.8668**	**0.88**	**0.8568**	**0.011636723**	0.754066667	**0.756**	0.7642	0.742	**0.011225566**
	**18**	**0.9374**	**0.9372**	**0.943**	**0.932**	**0.005502727**	0.867133333	0.864	0.884	0.8534	**0.015538769**
	**20**	**0.9524**	**0.9532**	**0.972**	**0.932**	**0.020011996**	0.832133333	0.8324	0.84	0.824	**0.008003333**
	**22**	**0.918833333**	**0.9165**	**0.935**	**0.905**	**0.015135499**	0.7821	0.7821	0.7821	0.7821	**1.35974E-16**
	24	**0.872333333**	0.869	0.889	0.859	**0.015275252**	0.7321	0.7321	0.7321	0.7321	**0**

KP														
	Time		O.D at 600nm											
			LB + Bacteria					200ppm						
	0		0.294866667	0.2946	0.296	0.294	0.00102632	0.29	0.285	0.29	0.295			0.2935
	2		0.482666667	0.493	0.485	0.47	0.011676187	0.313333333	0.3	0.31	0.33			0.375133333
	4		0.541333333	0.564	0.54	0.52	0.022030282	0.330333333	0.321	0.33	0.34			0.415833333
	6		0.671333333	0.694	0.67	0.65	0.022030282	0.35	0.34	0.35	0.36			0.469566667
	8		0.741333333	0.762	0.742	0.72	0.021007935	0.37	0.36	0.37	0.38			0.537233333
	10		0.821333333	0.842	0.82	0.802	0.020033306	0.39	0.38	0.39	0.4			0.5999
	12		0.908233333	0.9277	0.907	0.89	0.018880237	0.4	0.39	0.4	0.41			0.658533333
	14		1.017333333	1.112	1.02	0.92	0.096027774	0.429	0.41	0.437	0.44			0.717333333
	16		1.133466667	1.2668	1.0668	1.0668	0.115470054	0.459333333	0.43	0.468	0.48			0.754066667
	18		1.348	1.372	1.352	1.32	0.026229754	0.479666667	0.45	0.49	0.499			0.867133333
	20		1.435066667	1.4532	1.432	1.42	0.016811107	0.493333333	0.46	0.5	0.52			0.832133333
	22		1.145	1.165	1.14	1.13	0.018027756	0.403333333	0.39	0.4	0.42			0.7821
	24		0.9623	0.9869	0.96	0.94	0.023534443	0.311666667	0.29	0.3	0.345			0.7321

Comparison of Kpand Ko at different arsneite

	Time	KP + LB	AT-02+LB	KP 200ppm	AT-02 200ppm					
		**0.294866667**	0.295666667	0.29	0.2935	0.64698683	0.001176208			
	**0**	**0.482666667**	0.460666667	0.313333333	0.375133333					
	**2**	**0.541333333**	0.516733333	0.330333333	0.415833333					
	**4**	**0.671333333**	0.622666667	0.35	0.469566667					
	**6**	**0.741333333**	0.714133333	0.37	0.537233333					
	**8**	**0.821333333**	0.762733333	0.39	0.5999					
	**10**	**0.908233333**	0.839	0.4	0.658533333					
	**12**	**1.017333333**	0.893333333	0.429	0.717333333					
	**14**	**1.133466667**	0.996666667	0.459333333	0.754066667					
	**16**	**1.248**	1.14	0.479666667	0.867133333					
	**18**	**1.350666667**	1.2824	0.493333333	0.832133333					
	**20**	**1.145**	0.918833333	0.403333333	0.7821					
	**22**	**0.6387**	0.872333333	0.311666667	0.7321					

Table T7

Arsenic remediation by AT-02 biomass

	Avg				Stdev	t-test
0	99.63333333	99	101	98.9	1.18462371	
0.5	47.78666667	48.48	47.48	47.4	0.601775152	2.87151E-07
1	35.26666667	35	35	35.8	0.461880215	1.01421E-07
2	33.15	32	34.3	33.35	1.155782563	2.56707E-07

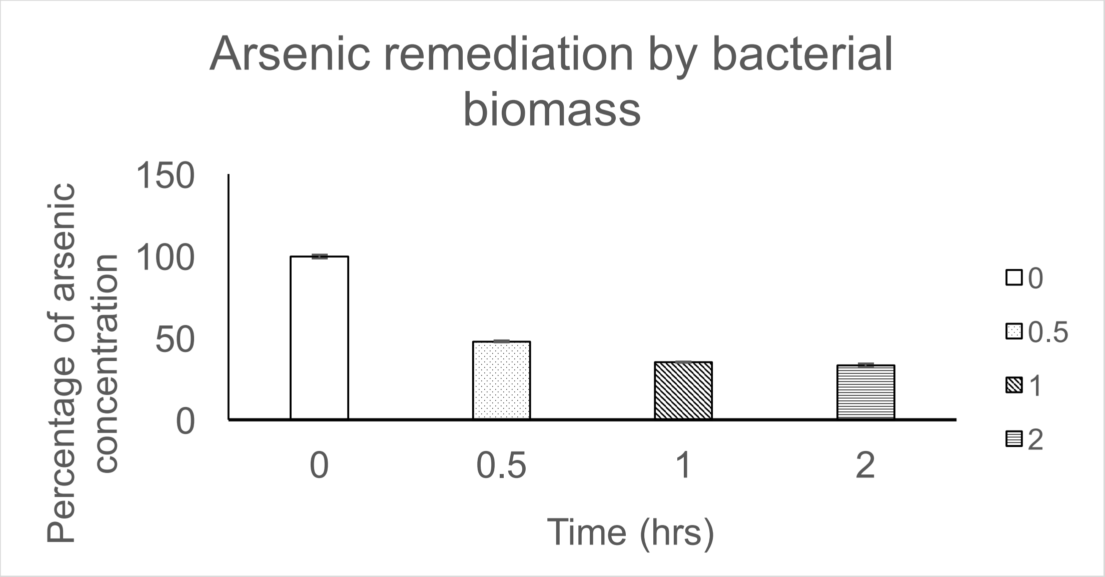

Arsenic remediation by bacterial biomass
Percentage of arsenic concentration
150
100
50
0
0
0.5
1
2
Time (hrs)
0
0.5
1
2

Table T8 Remediation efficiency of AT-02 biomass

	Ci-Cf/Cf*100		
	0	0	0
	50.03030303	53.99009901	50.97280081
	63.64646465	63.64646465	62.70182002
	66.67676768	67.03960396	65.17906977
Time	Remediation efficiency	Stdev	t-test
0	0	0	
0.5	51.66440095	2.068508911	1.70698E-06
1	63.3315831	0.545390829	3.66595E-09
2	66.29848047	0.986267094	3.26332E-08

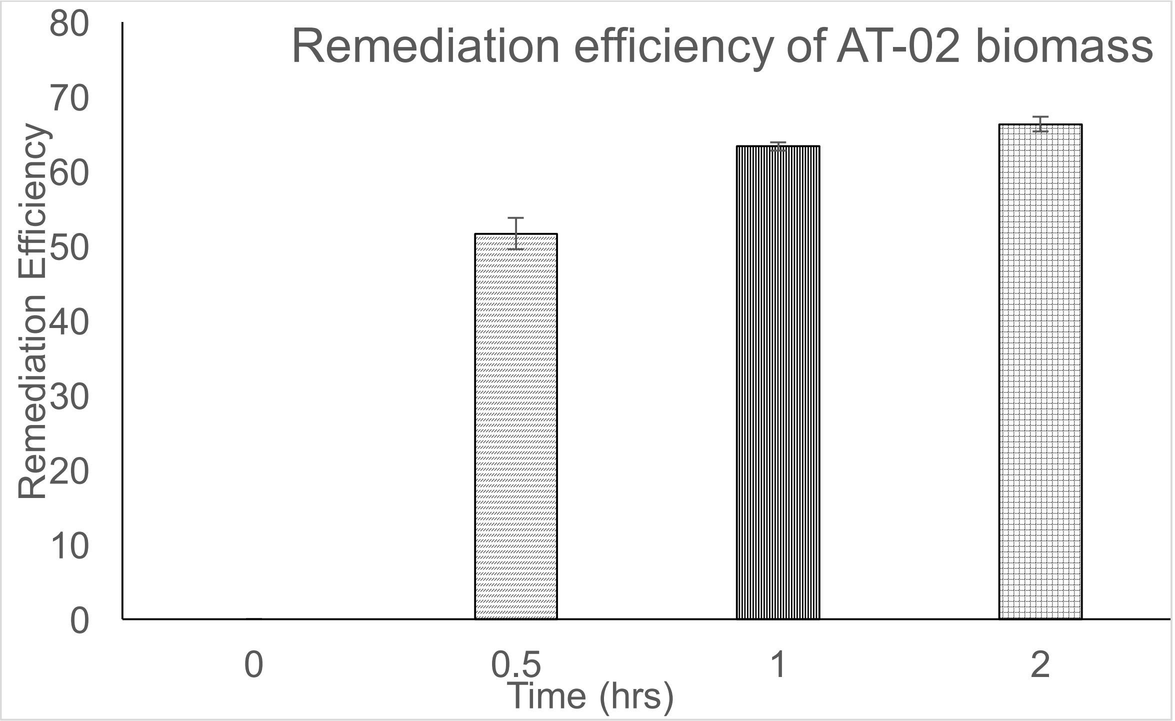

Remediation efficiency of AT-02 biomass
Remediation Efficiency
80
70
60
50
40
30
20
10
0
0
0.5
1
2
Time (hrs)

Table T9

Arsenic remediation by Kp biomass

	Avg	Treatment			Stdev		
0	100	99	101	100	0.816496581		
0.5	98.36021786	96.8	99.98	98.30065359	1.298912603	0.205189414	
1	95.63333333	95	95.9	96	0.449691252	0.002692603	
2	94.14575163	93	95.3	94.1372549	0.93899029	0.002649891	

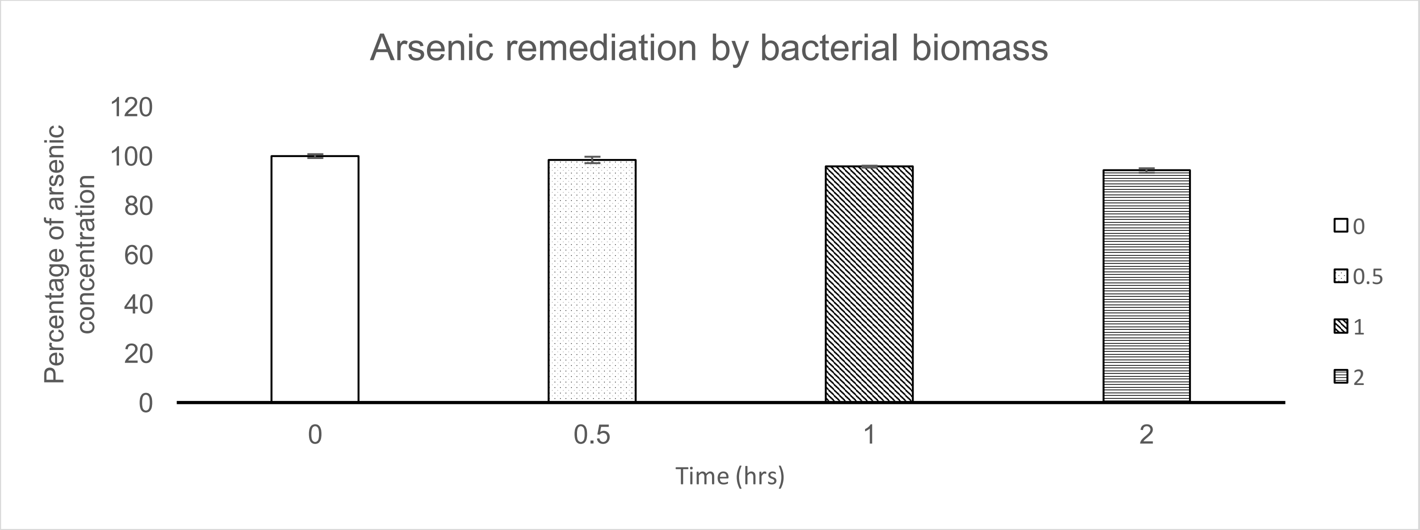
Arsenic remediation by bacterial biomass
Percentage of arsenic concentration
120
100
80
60
40
20
0
0
0.5
1
2
Time (hrs)
0
0.5
1
2

Table T10: Remediation efficiency of Kp(NK11) biomass

Ci-Cf/ci*100						
0	0	0				
1.639782135	1.222222222	1.699346405				
4.366666667	6.04950495	4				
5.060606061	6.643564356	5.862745098				
Time	Remediation efficiency				Stdev	t-test
0	0				0	
0.5	1.520450254				0.259984509	0.000534695
1	4.805390539				1.092921137	0.001595902
2	5.855638505				0.791503076	0.000213792

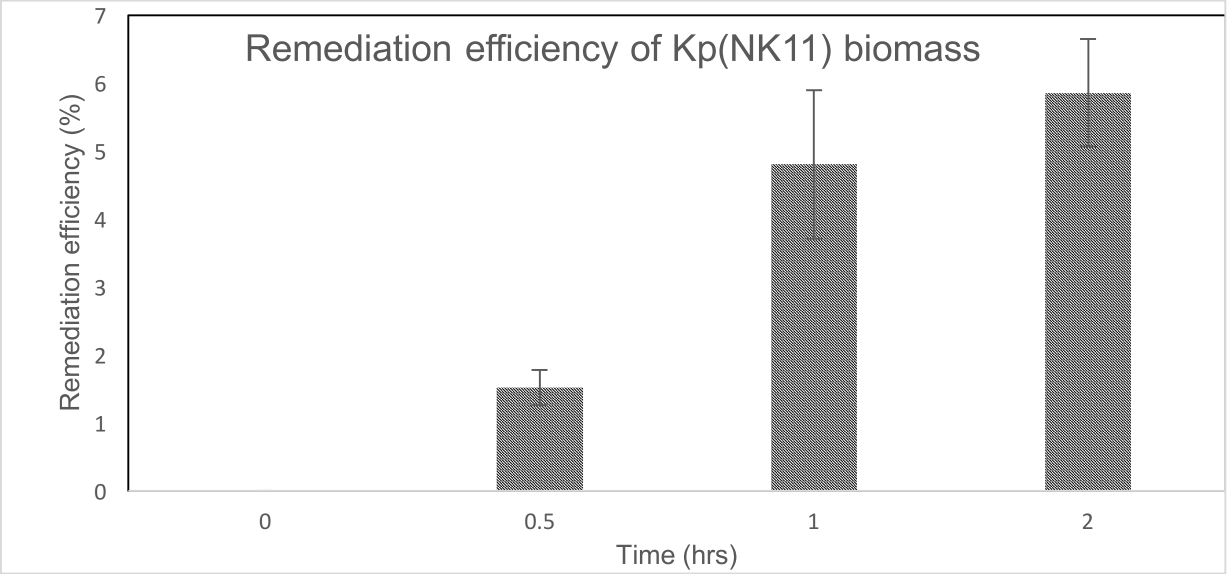

Remediation efficiency of Kp(NK11) biomass
Remediation efficiency (%)
0
1
2
3
4
5
6
7
0
0.5
1
2
Time (hrs)
