# Supplementary figures and images for: Arsenic efflux and bioremediation potential of Klebsiella oxytoca via the arsB gene
Source: PLoS One. 2025 Jan 29;20(1):e0307918. doi: 10.1371/journal.pone.0307918 (PMC11778763; doi:10.1371/journal.pone.0307918)

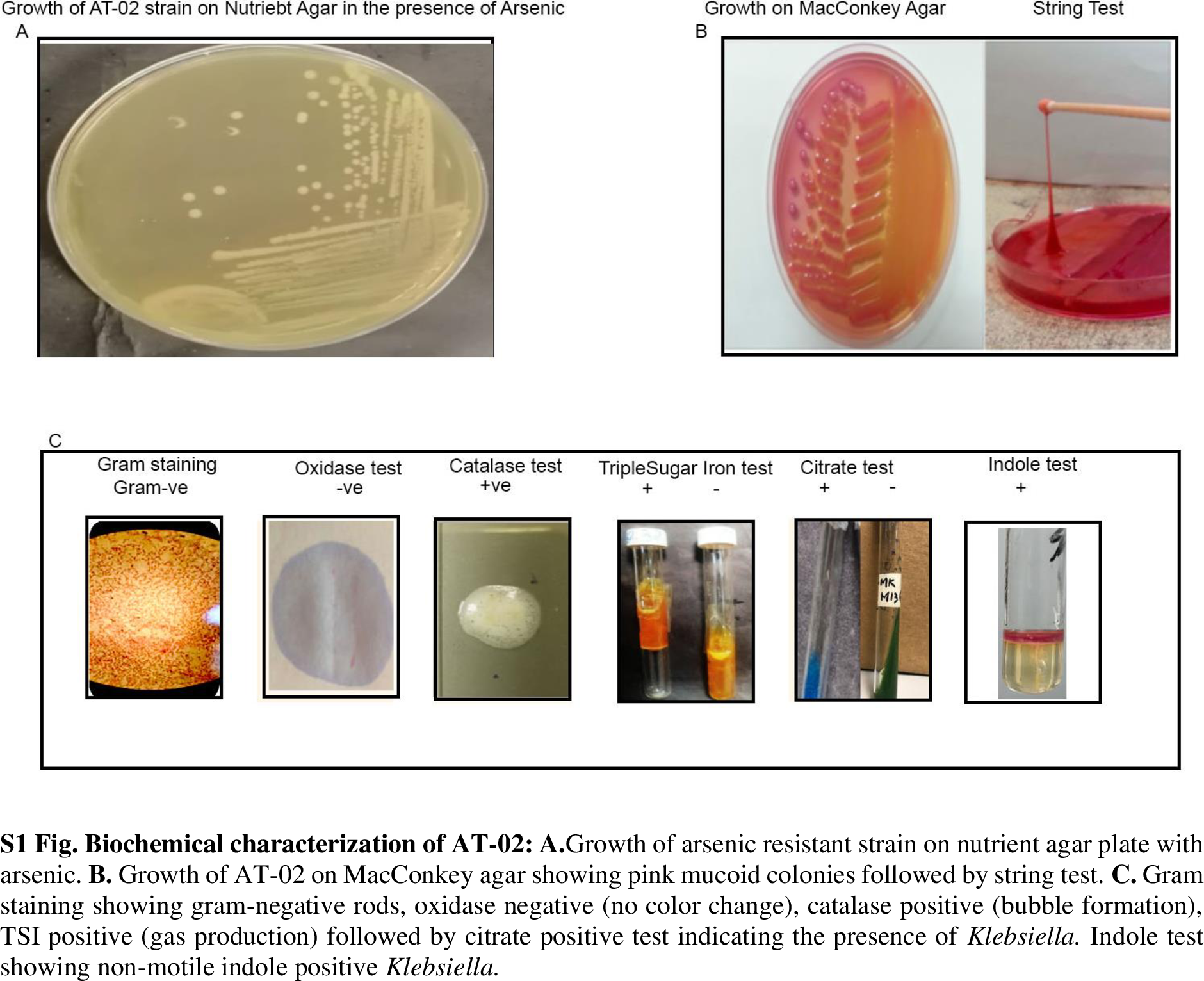

Supplement: S1 Fig — Biochemical characterization of AT-02: A. Growth of arsenic resistant strain on nutrient agar plate with arsenic. B. Growth of AT-02 on MacConkey agar showing pink mucoid colonies followed by string test. C. Gram staining showing gram-negative rods, oxidase negative (no color change), catalase positive (bubble formation), TSI positive (gas production) followed by citrate positive test indicating the presence of Klebsiella. Indole test showing non-motile indole positive Klebsiella. (TIF) [file pone.0307918.s002.tif]

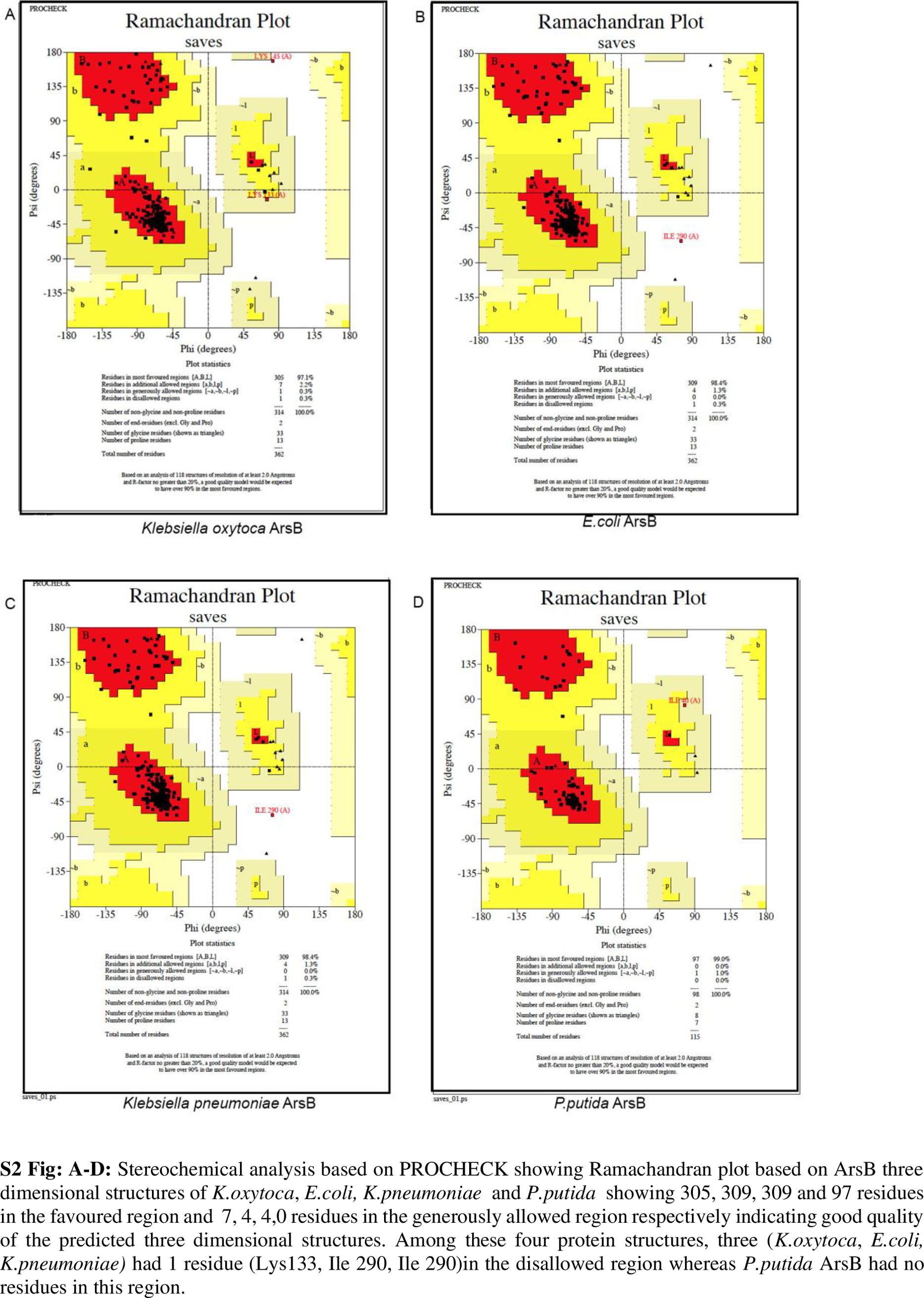

Supplement: S2 Fig — A-D: Stereochemical analysis based on PROCHECK showing Ramachandran plot based on ArsB three dimensional structures of K.oxytoca, E.coli, K.pneumoniae and P.putida showing 305, 309, 309 and 97 residues in the favoured region and 7, 4, 4,0 residues in the generously allowed region respectively indicating good quality of the predicted three dimensional structures. Among these four protein structures, three (K.oxytoca, E.coli, K.pneumoniae) had 1 residue (Lys133, Ile 290, Ile 290)in the disallowed region whereas P.putida ArsB had no residues in this region. (TIF) [file pone.0307918.s003.tif]

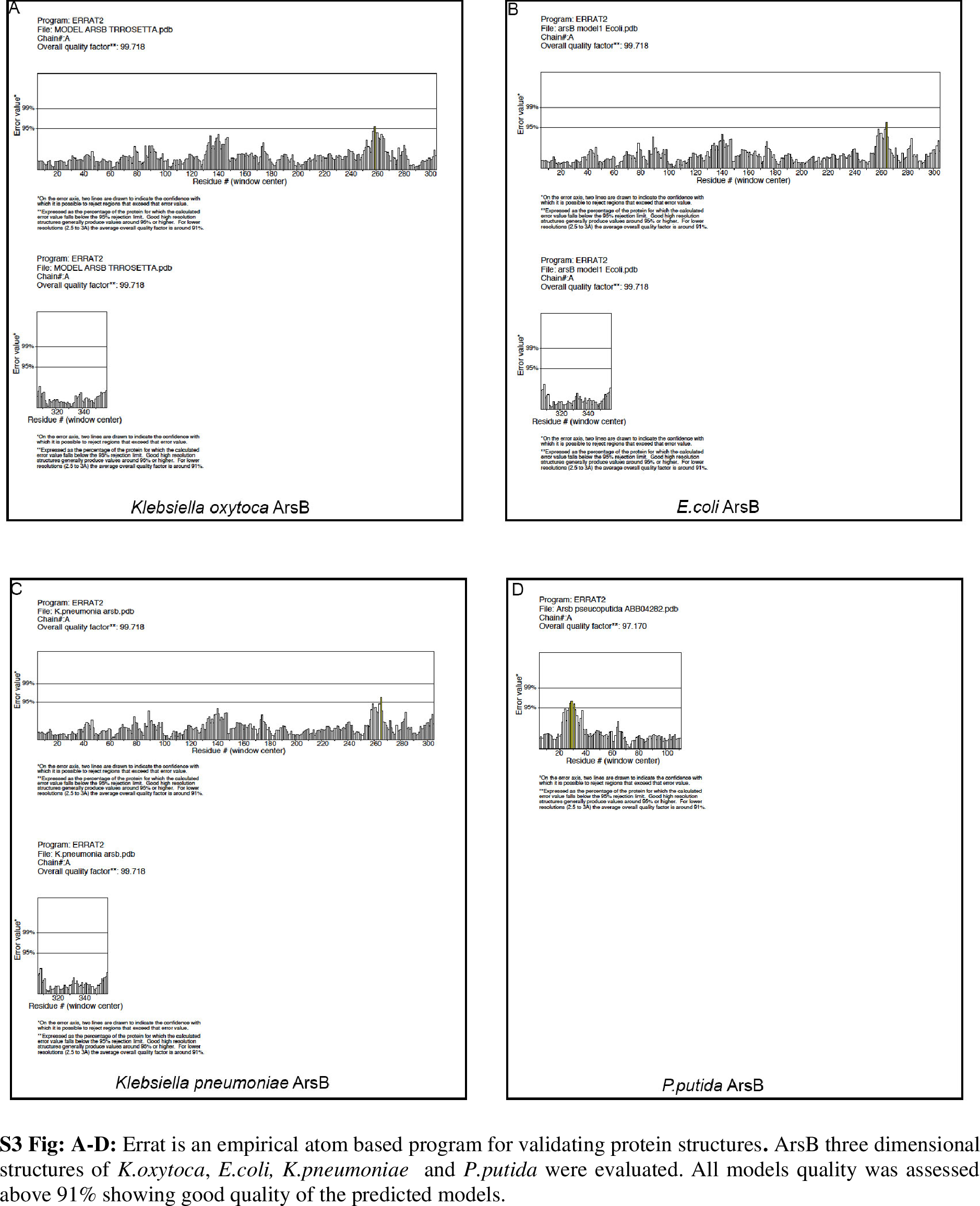

Supplement: S3 Fig — A-D: Errat is an empirical atom based program for validating protein structures. ArsB three dimensional structures of K.oxytoca, E.coli, K.pneumoniae and P.putida were evaluated. All models quality was assessed above 91% showing good quality of the predicted models. (TIF) [file pone.0307918.s004.tif]

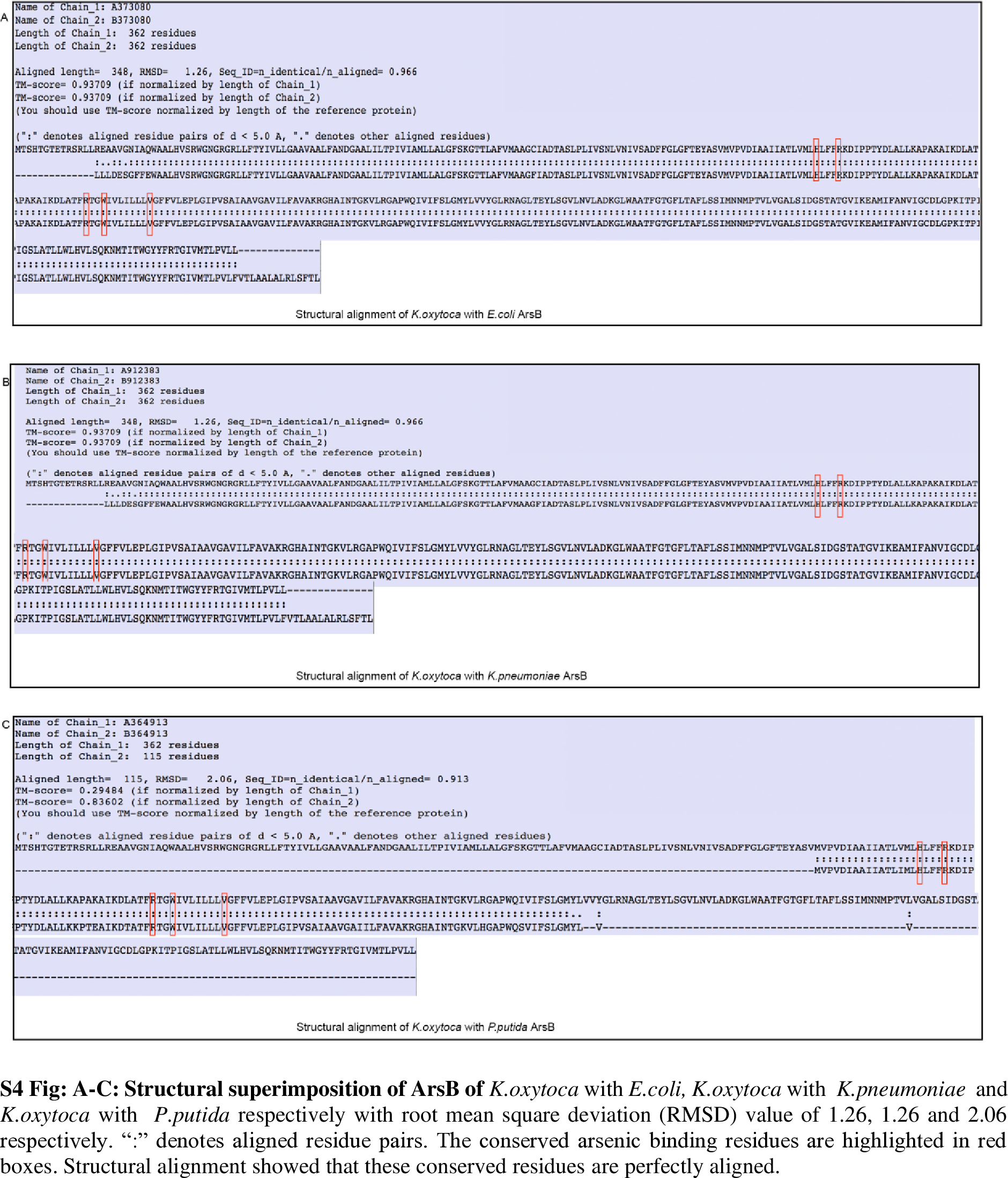

Supplement: S4 Fig — A-C: Structural superimposition of ArsB of K.oxytoca with E.coli, K.oxytoca with K.pneumoniae and K.oxytoca with P.putida respectively with root mean square deviation (RMSD) value of 1.26, 1.26 and 2.06 respectively. “:” denotes aligned residue pairs. The conserved arsenic binding residues are highlighted in red boxes. Structural alignment showed that these conserved residues are perfectly aligned. (TIF) [file pone.0307918.s005.tif]

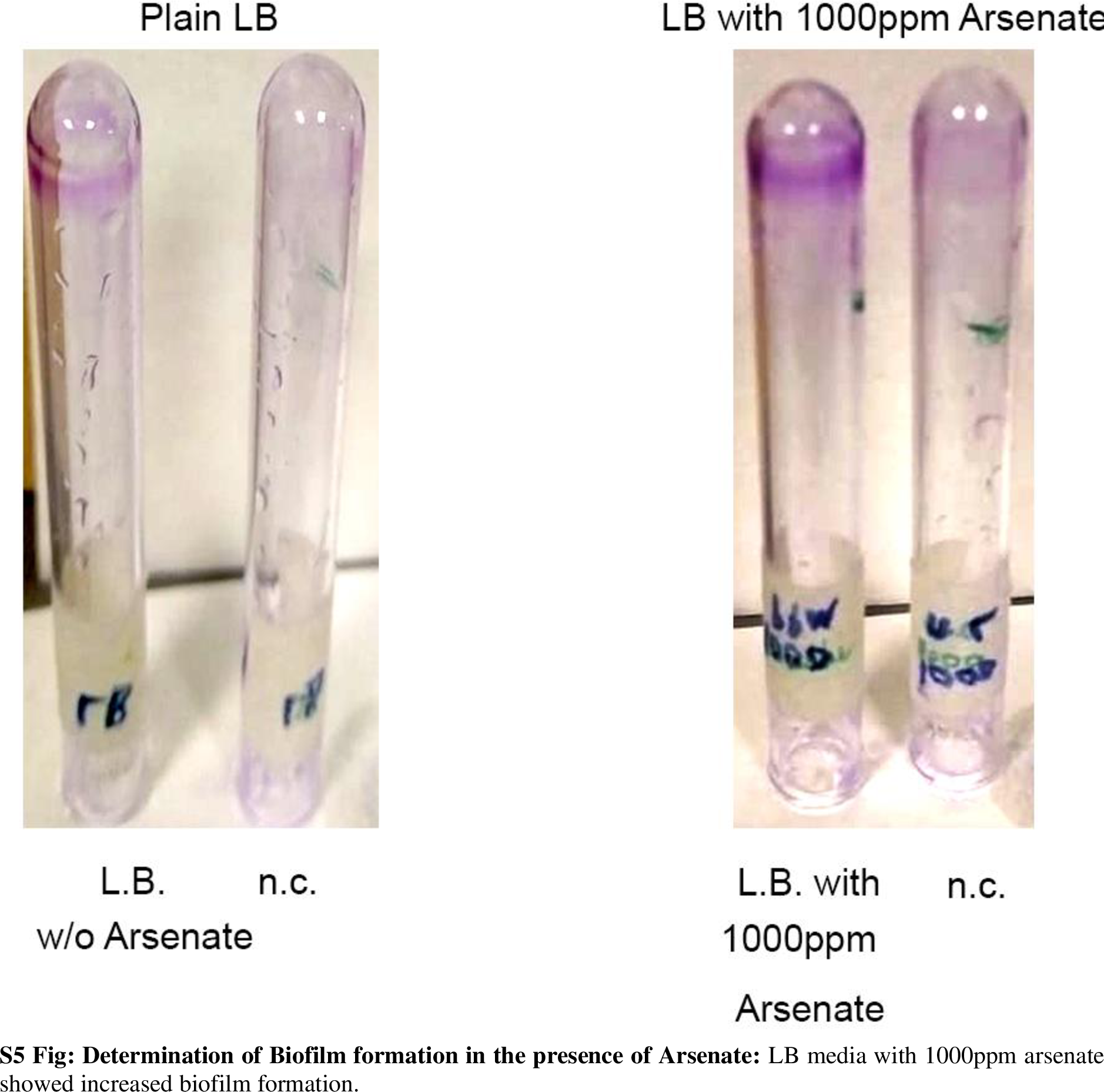

Supplement: S5 Fig — (TIF) [file pone.0307918.s006.tif]

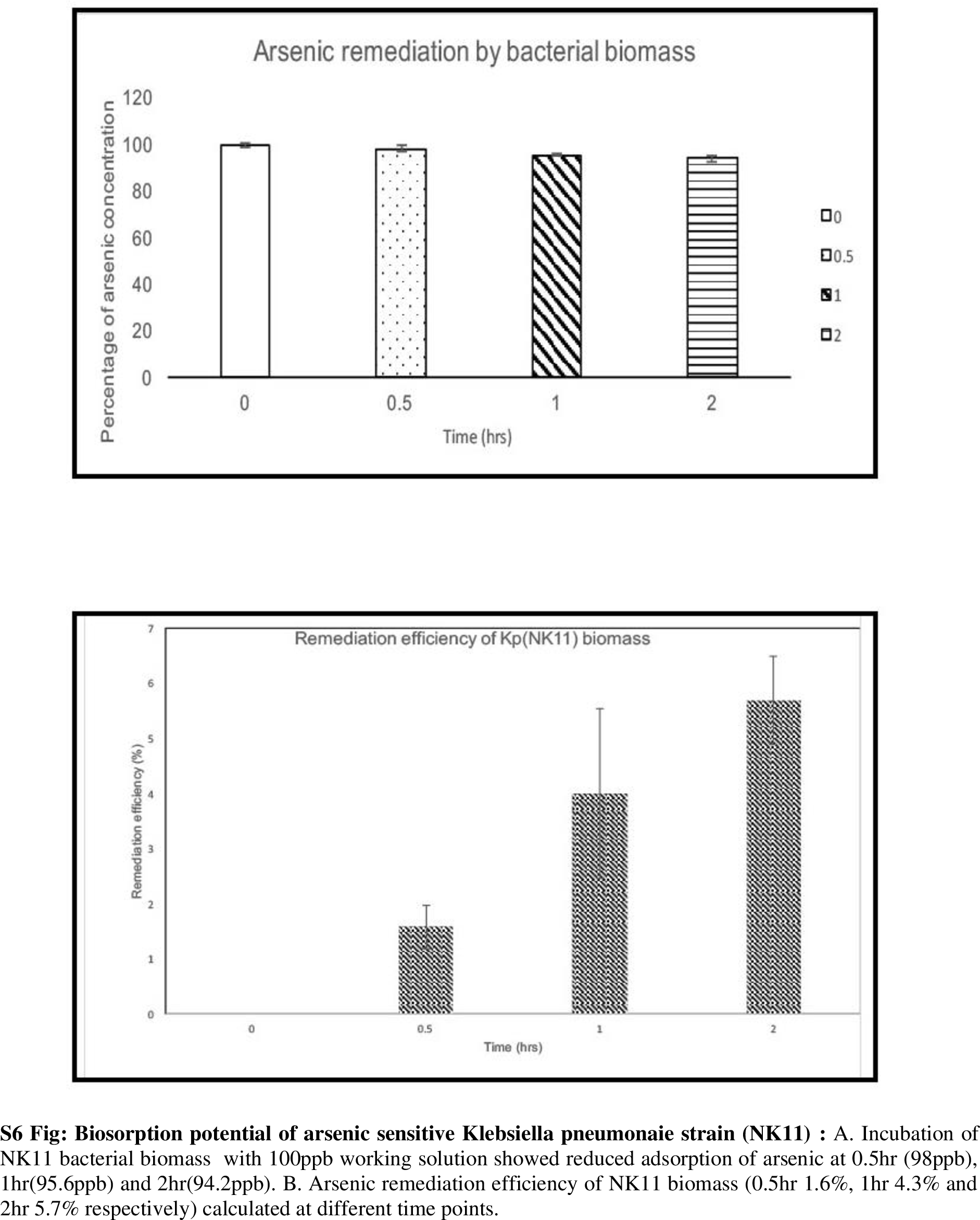

Supplement: S6 Fig — Biosorption potential of arsenic sensitive Klebsiella pneumonaie strain (NK11): A. Incubation of NK11 bacterial biomass with 100ppb working solution showed reduced adsorption of arsenic at 0.5hr (98ppb), 1hr(95.6ppb) and 2hr(94.2ppb). B. Arsenic remediation efficiency of NK11 biomass (0.5hr 1.6%, 1hr 4.3% and 2hr 5.7% respectively) calculated at different time points. (TIF) [file pone.0307918.s007.tif]
