# Supplementary material for: Arsenic efflux and bioremediation potential of Klebsiella oxytoca via the arsB gene
Source: PLoS One. 2025 Jan 29;20(1):e0307918. doi: 10.1371/journal.pone.0307918 (PMC11778763; doi:10.1371/journal.pone.0307918)
Supplement: S1 Table — (DOCX) [file pone.0307918.s018.docx]

# **Table 1:** Primers used for expression arsB expression analysis

| **Gene** | **Primer sequence** | **Product size**  **(bp)** | **Optimized temperature** |
| --- | --- | --- | --- |
| **arsB**  Forward  Reverse  **16SrRNA**  Forward  Reverse | ACTTACGACCTGGCTCTCCT  AATCGCACTGACCGGGATAC  CGG TTACCTTGTTACGACTT    CAGCAG CCGCGG TAATAC | 141 bp  180 bp | 60°C  61.5°C |
