# Supplementary material for: Arsenic efflux and bioremediation potential of Klebsiella oxytoca via the arsB gene
Source: PLoS One. 2025 Jan 29;20(1):e0307918. doi: 10.1371/journal.pone.0307918 (PMC11778763; doi:10.1371/journal.pone.0307918)
Supplement: S2 Table — (DOCX) [file pone.0307918.s019.docx]

**Table 2**: Physio-chemical properties of the water from Multan, Pakistan

| **Serial no.** | **Water quality parameters** | **Result** |
| --- | --- | --- |
| **Physical Parameter** | | |
| 1. | Color | Colorless |
| 2. | pH | 7.30 |
| 3. | Electrical conductivity (mS/cm) | 0.22 |
| 4. | Turbidity (NTU) | 7.30 |
| **Chemical Parameters** | | |
| 5. | Alkalinity (ppm) | 332 |
| 6. | Bicarbonate (ppm) | 332 |
| 7. | Calcium (ppm) | 101 |
| 8. | Carbonate (ppm) | BDL |
| 9. | Chloride (ppm) | 76 |
| 10. | Hardness (ppm) | 400 |
| 11. | Magnesium (ppm) | 36 |
| 12. | Potassium (ppm) | 6.6 |
| 13. | Sodium (ppm) | 76 |
| 14. | Sulfate (ppm) | 120 |
| 15. | TDS (ppm) | 574 |
| **Trace and Ultra-Trace Elements** | | |
| 16. | Arsenic (ppb) | 184 |
