# Supplementary material for: Arsenic efflux and bioremediation potential of Klebsiella oxytoca via the arsB gene
Source: PLoS One. 2025 Jan 29;20(1):e0307918. doi: 10.1371/journal.pone.0307918 (PMC11778763; doi:10.1371/journal.pone.0307918)
Supplement: S3 Table — (DOCX) [file pone.0307918.s020.docx]

**Table 3:** Arsb arsenic binding residues mutation consequence prediction by PROVEAN

| **Sr.No.** | **Variant** | **PROVEAN score** | **Prediction**  **(cutoff**  **-2.5)** |
| --- | --- | --- | --- |
| 1 | H142A | -1.512 | Neutral |
| 2 | R146A | -5.274 | Deleterious |
| 3 | R172A | -3.332 | Deleterious |
| 4 | W175A | -12.072 | Deleterious |
| 5 | V183A | -2.138 | Neutral |
