# Supplementary material for: Arsenic efflux and bioremediation potential of Klebsiella oxytoca via the arsB gene
Source: PLoS One. 2025 Jan 29;20(1):e0307918. doi: 10.1371/journal.pone.0307918 (PMC11778763; doi:10.1371/journal.pone.0307918)
Supplement: S4 Table — (DOCX) [file pone.0307918.s021.docx]

**Table 4:** Arsb arsenic binding residues mutation consequence prediction by DDMUT on protein stability

| **Sr.No.** | **Variant** | **ΔΔG** |
| --- | --- | --- |
| 1 | H142A | -0.31 |
| 2 | R146A | -0.06 |
| 3 | R172A | -0.04 |
| 4 | W175A | -1.57 |
| 5 | V183A | -0.30 |
